# Mechanical response and energy dissipation law of double-fractured sandstone under dynamic load

**DOI:** 10.1038/s41598-025-20378-y

**Published:** 2025-10-17

**Authors:** Qinghai Zhang, Xiaoliang Xu, Lihua Wu, Jianlin Li, Quan Shi, Delin Tan

**Affiliations:** 1 Key Laboratory of Geological Hazards on Three Gorges Reservoir Area, Ministry of Education, Three Gorges University, Yichang, 443002 Hubei China; 2 Suihua University, Suihua, 152001 Heilongjiang China; 3 State Key Laboratory of Disaster Prevention and Mitigation of Explosion and Impact, The Army Engineering University of PLA, Nanjing, 210007 Jiangsu China

**Keywords:** SHPB test, Dynamic strength, Crack initiation stress, Energy dissipation, Fractal dimension, Average fragment size, Civil engineering, Natural hazards, Petrology, Geodynamics

## Abstract

To study the dynamic mechanical response and energy dissipation law of fractured sandstone, an SHPB device combined with a high-speed camera system was used to carry out impact compression tests with seven rock bridge angle specimens. The dynamic strength characteristics of fractured sandstone and the crack initiation stress at the fracture tip was explored. The fractal characteristics of debris were quantitatively described, and the relationship between energy dissipation and fractal dimension and average debris size was analyzed. In addition, a significant rate effect between rock angle and dynamic load was observed with peak stress degradation value of the sample been significantly affected by the rock angle. The results show that the strain value of peak stress increases first and then decreases with rock angle increase; the peak stress values of the intact specimen show a sensitive change characteristic, and the prefabricated fractures significantly weaken the peak stress. The maximum and minimum rock angles are 0°and 90°, respectively. Under low dynamic load and small rock bridge angle, cracks are single and dispersed; under high dynamic load and large rock bridge angle, crack types are diverse, showing a composite failure of shear and tension. Dynamic load and rock bridge angle have a significant impact on the stress and deterioration performance of the samples, especially under higher dynamic load and larger rock bridge angle, where the stress deterioration of the samples is more pronounced. The crack initiation stress at the fracture tip decreases with rock angle increase and is lower than the peak stress. There are significant differences in the initiation time of cracks (damage velocity) at the fracture tip. The damage velocity is significantly affected by dynamic load; there is a significant rate effect between the incident energy and the reflected energy and the dynamic load. The energy dissipation rate and fractal dimension increase with dynamic load; the energy dissipation rate has an opposite relationship with the exponential curve of fractal dimension and average fragment size, and the 60° specimen shows more sensitive fractal variation characteristics.

## Introduction

**Fig. 1:**
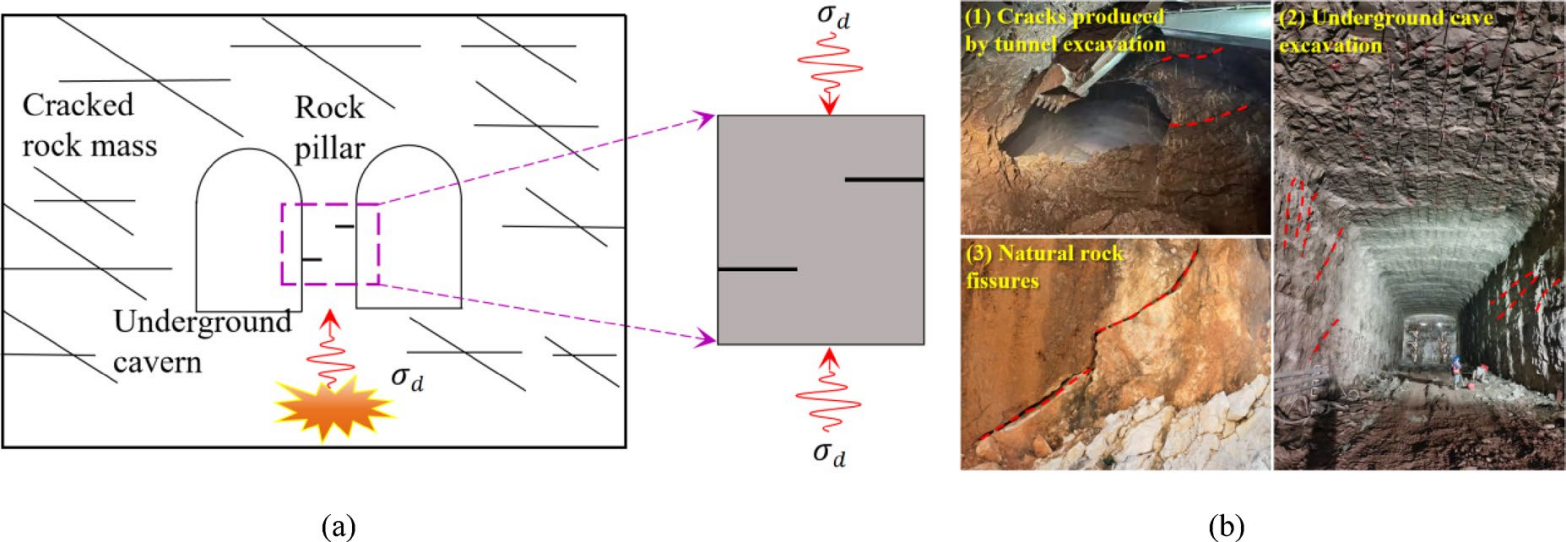
Schematic diagram and field diagram of underground rock mass structure under static-dynamic coupling action. **(a)** Schematic diagram of underground structure. **(b)** Map of the natural fragmented rock mass and underground space.

 Rock mass is a complex material composed of one or more minerals. It contains a large number of naturally occurring defects, such as fractures, faults, and joints. Defects significantly weaken rock mass strength and structure stability^1–3^. Although there are certain rules for the shape or distribution of cracks, the mechanical properties of rock mass under the influence of their interaction are complex and changeable. In line with rock fracture theories inherent discontinuities in rockmass constitute points of crack initiation and propagation when applied stresses exceed rockmass strength^4–9^. This will destroy the engineering rock mass structure and even cause disaster^10–13^, The stress redistribution of rock mass during the excavation unloading process of underground engineering causes existing cracks expand and emerging ones to start. The stability and safety of rock mass engineering under dynamic loads may be threatened by the significant variations in the dynamic mechanical properties, failure characteristics, and energy dissipation of fractured rock mass, especially during dynamic loads such as blasting, explosion, and rock burst^14–16^, (Fig. 1). Therefore, it is of significance to conduct in-depth research on the dynamic characteristics of defective rock mass and reveal the dynamic mechanical properties and energy dissipation law of fractured rock mass under dynamic load.

In recent years, domestic and foreign scholars have carried out extensive research on the mechanical properties and crushing energy of fractured rock mass (including rock, coal samples, similar materials and similar rock mass). The rock mass with different rock bridges, dip angles, lengths, crossings, numbers and arrangements of cracks is taken as the research object. Direct shear^17–19^, uniaxial^20–22^ and triaxial^23,24^ tests were carried out to study the mechanical properties, energy dissipation and failure characteristics of the response, and fruitful research results were obtained. Dynamic loads such as blasting and rock bursts are common in underground engineering. It shows different characteristics from static loads. The propagation of stress waves, the development of rock mass deformation and the dynamic evolution of cracks are higher than those under static loading conditions^25^.

At present, for the study of the dynamic mechanical properties of fractured rock samples, the impact compression test is mainly carried out by SHPB equipment. Li et al.^26^ conducted impact compression tests on prefabricated single-fractured cylindrical sandstone samples with 7 different loading angles. It is concluded that different inclination angles have a significant effect on dynamic compressive strength, dynamic strain and dynamic elastic modulus. Wen et al.^27^ took the rectangular plate red sandstone as the research object, and carried out the impact compression test combined with the high-speed camera system. The results show that with the increase of strain rate, different fracture dip angles affect the crack propagation mode, and the tensile crack evolves into the shear crack. When the fracture dip angle is constant, the dynamic strength shows obvious strain rate effect, and the fracture dip angle has a significant effect on the degree of rock impact fragmentation and fractal dimension. Sun et al.^28^ conducted an impact compression test on the prefabricated single-fracture cylindrical coal-rock combination. It was found that when the fracture is located in the coal body or the joint surface, the proportion of crushing energy consumption and the crushing energy consumption density of the fracture combination specimen are small, and when the fracture is located in the rock mass, it is larger. The existence of the fracture affects the crushing characteristics of the combination specimen. Wang et al.^29^ used prefabricated single-crack cuboid rock materials to carry out impact compression tests. It was concluded that the failure mode of rock samples with prefabricated cracks was mainly tensile-shear failure, and the crack angle had a certain influence on the dynamic strength. The correlation between the dynamic initiation toughness of composite cracks and the classical criterion under different loading rates and different joint angles was analyzed. Li et al.^30^ conducted impact compression tests on single-crack cylindrical granite specimens with different dip angles. It was found that the crack dip angle had a significant effect on the dynamic strength parameters and energy absorption rate. This would change the failure mode of specimens with cracks. The crack initiation angle and crack initiation stress changed in the form of “M” and “W” with the increase of the prefabricated crack angle, respectively. However, there are relatively few experimental studies on the dynamic characteristics of multi-fractured rock mass. Taking prefabricated double-fractured and multi-fractured sandstone as the research object, the relationship between impact pressure and dynamic compressive strength of double-fractured sandstone is discussed. The failure mode of the sample and the mutual transformation of the failure modes of different fracture angles^31,32^. The static and dynamic composite loading test of multi-crack sandstone was carried out by using improved SHPB equipment. It was found that the number of cracks had obvious rate effects on dynamic strength, total strength and deformation modulus. It was positively correlated with the average fragment size and fractal dimension^33,34^. Wu et al.^35^ Combined with DIC technology, the dynamic compression test of concrete specimens containing five kinds of single cracks with different inclination angles was carried out. It is concluded that different prefabricated crack angles and lengths have significant effects on the dynamic mechanical properties, wave dissipation and amplitude of the specimens. The failure mode is closely related to the length of the prefabricated crack, and the correlation with crack width is weak. Zhao et al.^36^ took coal samples with different bedding angles as research objects, combined with high-speed camera system and digital speckle image analysis method, and carried out an impact compression test. The results showed that impact velocity and surface angle had an obvious influence on coal samples’ dynamic tensile failure characteristics. The greater the impact velocity, the greater the dynamic tensile strength. When the bedding dip angle is parallel or non-vertical to the loading direction, the coal sample is more likely to exhibit tensile failures. On the contrary, the coal sample showed matrix tensile and bedding shear failures.

In summary, most of the current research focused on the static test of specimens with different crack forms. Research objects include rock, coal samples, similar materials and rock-like materials. These studies have produced specimens with different rock bridges, fracture angles, lengths, numbers and arrangement modes. They have discussed the static mechanical properties, energy dissipation and crushing characteristics of fractured rock masses, and initially formed a relatively complete theoretical framework. However, when it comes to underground engineering, dynamic load is one of the key factors that causes disasters. Although existing research has carried out a more in-depth analysis of the static mechanical properties of fractured rock mass, the dynamic mechanical properties, energy dissipation and crushing characteristics of fractured rock mass under dynamic load, especially the influence of different rock bridge angles on the mechanical response in multi-fractured rock mass, have received relatively little attention. Although some studies have examined the dynamic mechanical response of rocks with multiple fractures, most have focused on relatively simple crack configurations. They lack a systematic analysis of the complexity of multi-fractured rock masses, crack interactions, and the influence of rock-bridge angle. To address this gap, this study uses a split Hopkinson pressure bar (SHPB) apparatus to conduct impact compression tests on sandstone specimens containing either edge-offset double cracks or parallel double cracks, investigating their dynamic mechanical behavior under different levels of dynamic loading. By integrating high-speed imaging to analyze the crack-initiation stress at crack tips, the study reveals the energy-evolution characteristics of fractured sandstone and offers an in-depth discussion of the dynamic response mechanisms of multi-fractured rock masses, thereby advancing research on fractured rock dynamic mechanical properties.

## Test methods

### Specimen Preparation and test scheme

**Fig. 2:**
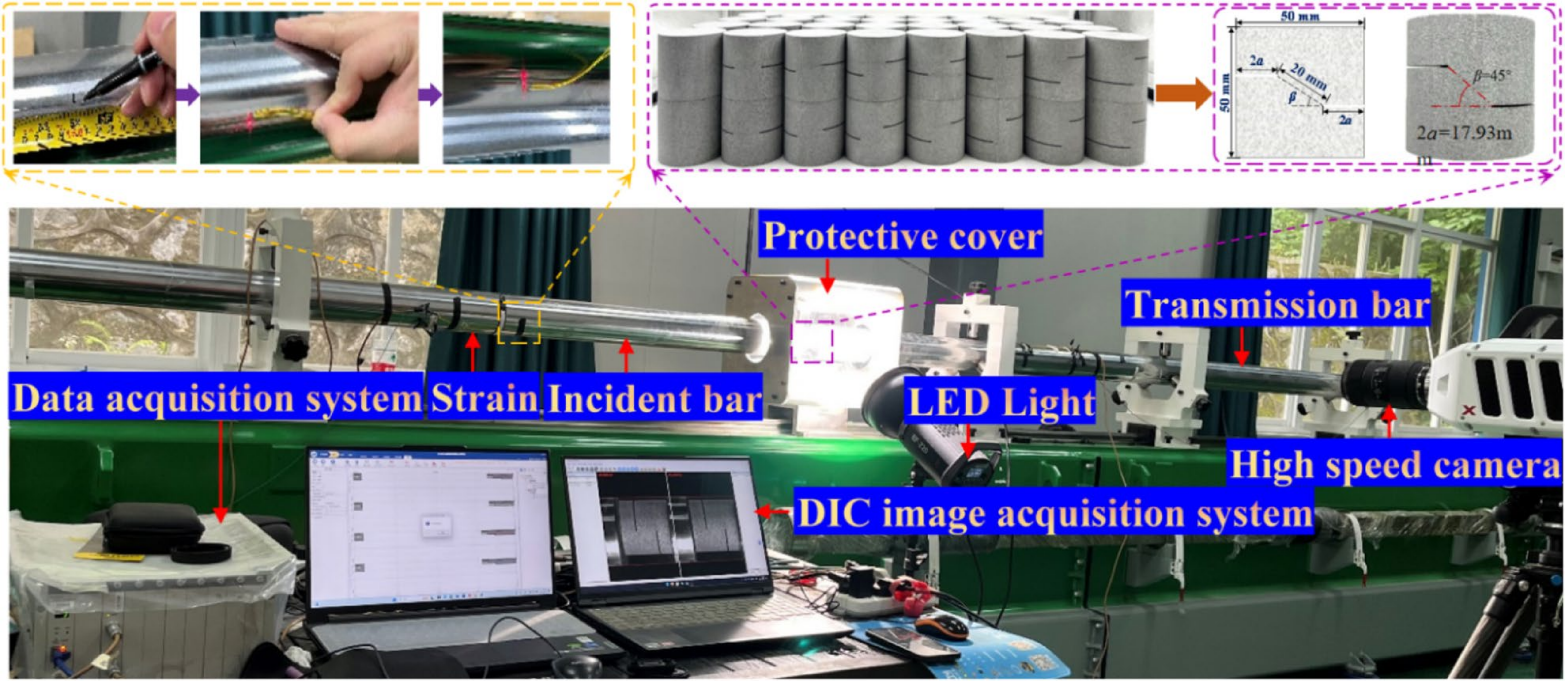
SHPB test field diagram.

In this study, the specimen is sandstone. According to the International Society of Rock Mechanics (ISRM) recommended test procedures^37^ and the Rock Dynamic Characteristics Test Procedure^38^, a standard cylinder with a diameter of D = 50 mm, a height of H = 50 mm, a flatness of less than 0.02 mm, an axis deviation of no more than 0.1% rad, and a vertical smooth outer surface is made. To reduce the influence of anisotropy, the same sandstone was selected for coring in the same direction. Different rock bridge angles (rock angles) are 0°, 15°, 30°, 45°, 60°, 75° and 90° respectively, and intact specimens are added for comparison^39–42^. Specimens and fracture structure diagrams are shown (Fig. 2). In this study, the SHPB device was used. The striker bar diameter is 100 mm and the length is 600 mm. According to the law of conservation of energy, the impact pressure value is set too small. Six different impact loads (dynamic loads) are designed in the test: 0.10 MPa, 0.15 MPa, 0.20 MPa, 0.25 MPa, 0.30 MPa and 0.35 MPa, respectively. The corresponding average impact velocities are 3.40 m/s, 5.42 m/s, 7.39 m/s, 9.31 m/s, 11.41 m/s and 13.30 m/s, respectively. Specimen NO.: YS-20-*β* represents the rock bridge length of the specimen is 20 mm and the horizontal angle *β*.

The test was carried out in the SHPB device of the Key Laboratory of Geological Disasters of the Ministry of Education in the Three Gorges Reservoir Area. The main components of this system are the air emission system, the equipment console, and the high-performance dynamic strain acquisition instrument DH8302. The bar material of the test device is 60Si2Mn alloy steel, the yield strength is 1230 MPa, the diameter is 100 mm, the longitudinal wave velocity is 5122.7 m/s, the Poisson’s ratio is 0.28, the elastic modulus is 206 GPa, the density is 7850 kg.m^−3^, the incident bar is 4.5 m, the transmission bar is 3.5 m, and the absorption bar is 2.0 m, the structure and field test are shown (Fig. 2).

The instantaneous strain is measured by a strain gauge mounted on the incident bar and the transmission bar. When pasting the strain gauge, it is necessary to pay attention to polishing the surface of the incident bar and the transmission bar. The fast solid adhesive was placed at 0.8 m (twice the length of the bullet) at the interface between the specimen and the bar. The specimen’s dynamic strain is measured by the DHHP-20 super dynamic data acquisition instrument, and the maximum sampling frequency is 10 MHz.

### Energy theory and dynamic equilibrium

**Fig. 3:**
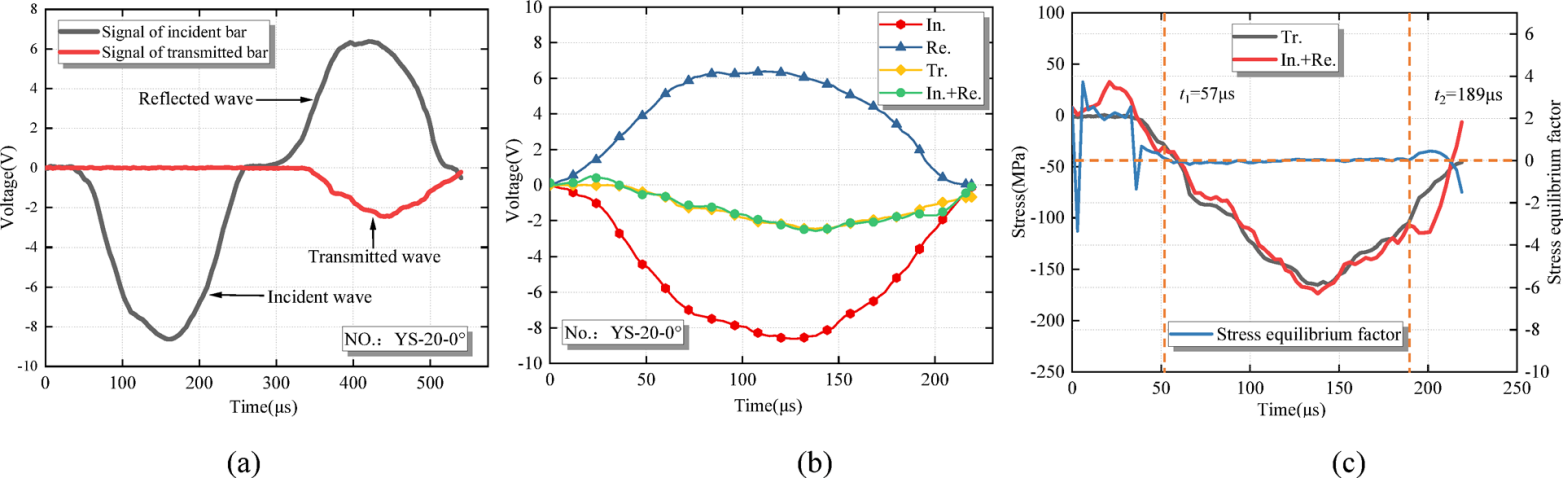
Verification of dynamic stress balance in SHPB test. **(a)** Original voltage pulse signal. **(b)** Dynamic balance verification. **(c)** Dynamic balance factor.

To ensure the validity of the test results, it is necessary to ensure the dynamic balance and stability of the specimen during the test. This should conform to the stress balance conditions at both ends of the specimen and the one-dimensional stress wave hypothesis theory. When the air pressure driven transmitting bar impacts the incident bar, the incident strain $\:{\varepsilon\:}_{\text{I}}\left(t\right)$ is generated on the incident bar, the reflection strain $\:{\varepsilon\:}_{\text{R}}\left(t\right)$ is generated on the surface of the specimen and the incident bar, and the transmission strain $\:{\varepsilon\:}_{\text{T}}\left(t\right)$ is produced on the surface of the transmission bar, which propagates along the incident bar and the transmission bar respectively (Fig. 3**(a)**). It is assumed that the elastic wave propagation velocity of the bar is $\:{C}_{0}$, the length and cross sectional area of the specimen are $\:{L}_{\text{s}}$ and $\:{A}_{\text{s}}$, and the elastic modulus and cross-sectional area of the bar are $\:{E}_{0}$ and $\:{A}_{0}$. Therefore, according to the uniformity assumption, the axial load $\:P\left(t\right)$, average stress $\:\sigma\:\left(t\right)$, strain $\:\varepsilon\:\left(t\right)$ and strain rate $\:\dot{{\upepsilon\:}}\left(t\right)$ at both ends of the specimen are calculated^43,44^. The formula is as follows:

1 $$\:P\left(t\right)={\text{A}}_{0}{\text{E}}_{0}\left[{\varepsilon\:}_{\text{I}}\right(t)+{\varepsilon\:}_{\text{R}}(t\left)\right]={\text{A}}_{0}{\text{E}}_{0}{\varepsilon\:}_{\text{T}}\left(t\right)$$

2 $$\:\left\{\begin{array}{c}\sigma\:\left(t\right)=\frac{{A}_{0}{E}_{0}}{{2A}_{\text{S}}}\left[{\varepsilon\:}_{\text{T}}\right(t)+{\varepsilon\:}_{\text{R}}(t)+{\varepsilon\:}_{\text{I}}(t\left)\right]\\\:\varepsilon\:\left(t\right)=\frac{{C}_{0}}{{L}_{\text{S}}}{\int\:}_{0}^{t}\left[{\varepsilon\:}_{\text{I}}\right(t)-{\varepsilon\:}_{\text{R}}(t)-{\varepsilon\:}_{\text{T}}(t\left)\right]dt\\\:\dot{\varepsilon\:}\left(t\right)=\frac{{C}_{0}}{{L}_{\text{S}}}\left[{\varepsilon\:}_{\text{I}}\right(t)-{\varepsilon\:}_{\text{R}}(t)-{\varepsilon\:}_{\text{T}}(t\left)\right]\end{array}\right.$$

Crack propagation and failure are energy dissipation and absorption processes. These processes are directly related to the number and surface area of primary and new fractures, and the main energy source is incident energy. According to the research results of Wang et al.^45^ and Zhou et al.^46^, the formulas for incident energy, reflection energy, transmission energy, dissipation energy, energy dissipation rate, and energy dissipation density are calculated as follows:

3 $$\:{W}_{\text{I}}={E}_{0}{C}_{0}{A}_{0}{\int\:}_{0}^{t}{{\upepsilon}}_{\text{I}}^{\text{2}}\left(t\right)dt$$

4 $$\:{W}_{\text{R}}={E}_{0}{C}_{0}{A}_{0}{\int\:}_{0}^{t}{{\upepsilon}}_{\text{R}}^{\text{2}}\left(t\right)dt$$

5 $$\:{W}_{\text{T}}={E}_{0}{C}_{0}{A}_{0}{\int\:}_{0}^{t}{{\upepsilon}}_{\text{T}}^{\text{2}}\left(t\right)dt$$

6 $$\:{W}_{\text{L}}={W}_{\text{I}}-({W}_{\text{T}}+{W}_{\text{R}})$$

7 $$\:{E}_{\text{A}}=\frac{{W}_{\text{L}}}{{W}_{\text{I}}}\times\:100\%$$

8 $$\:{E}_{\rho\:}=\frac{{W}_{\text{L}}}{V}$$

The formula: $\:{W}_{\text{I}}$, $\:{W}_{\text{R}}$, and $\:{W}_{\text{T}}$ are the energy carried by incident wave, reflected wave, and transmitted wave, respectively, J; $\:{W}_{\text{L}}$ is dissipated energy, J, ignoring the energy loss of the bar and the specimen surface; $\:{E}_{\text{A}}$ is the energy dissipation rate, %; $\:{E}_{{\uprho\:}}$ is the energy dissipation density, J/cm^3^; $\:\text{V}$ is the specimen volume, cm^3^.

According to the one-dimensional stress wave hypothesis theory, the stress at both ends of the specimen needs to be dynamically balanced before failure. To verify whether the two ends of the specimen can maintain a dynamic balance, the stress at both ends is calculated and compared. In the whole process of transmission wave fluctuation, the transmission wave should be approximately equal to the sum of the incident wave and the reflected wave (Fig. 3**(b)**). In addition, the dynamic stress balance factor $\:\left({\upvarphi\:}\right)$ is introduced to quantitatively analyze the stress equilibrium state at both ends of the specimen. The formula is as follows^47^:

9 $$\:{\upvarphi\:}=\frac{2\left[{{\upsigma\:}}_{\text{I}}\right(t)+{{\upsigma\:}}_{\text{R}}(t)-{{\upsigma\:}}_{\text{T}}(t\left)\right]}{{{\upsigma\:}}_{\text{I}}\left(t\right)+{{\upsigma\:}}_{\text{R}}\left(t\right)+{{\upsigma\:}}_{\text{T}}\left(t\right)}$$

Figure 3**(c)** shows that the dynamic stress balance factor $\:\left({\upvarphi\:}\right)$ between 57 us and 189 us is about 0. It is considered that a dynamic balance is realized at both ends of the specimen during this time period. The axial loads at both ends are approximately equal. Quasi-static stress analysis can be performed, and inertia is neglected. In order to save space, only the original voltage pulse signal and dynamic balance check of the specimen with a rock angle of 0° and a dynamic load of 0.15 MPa are shown (Fig. 3).

## Test results and mechanical properties

### Full stress-strain relationship

**Fig. 4:**
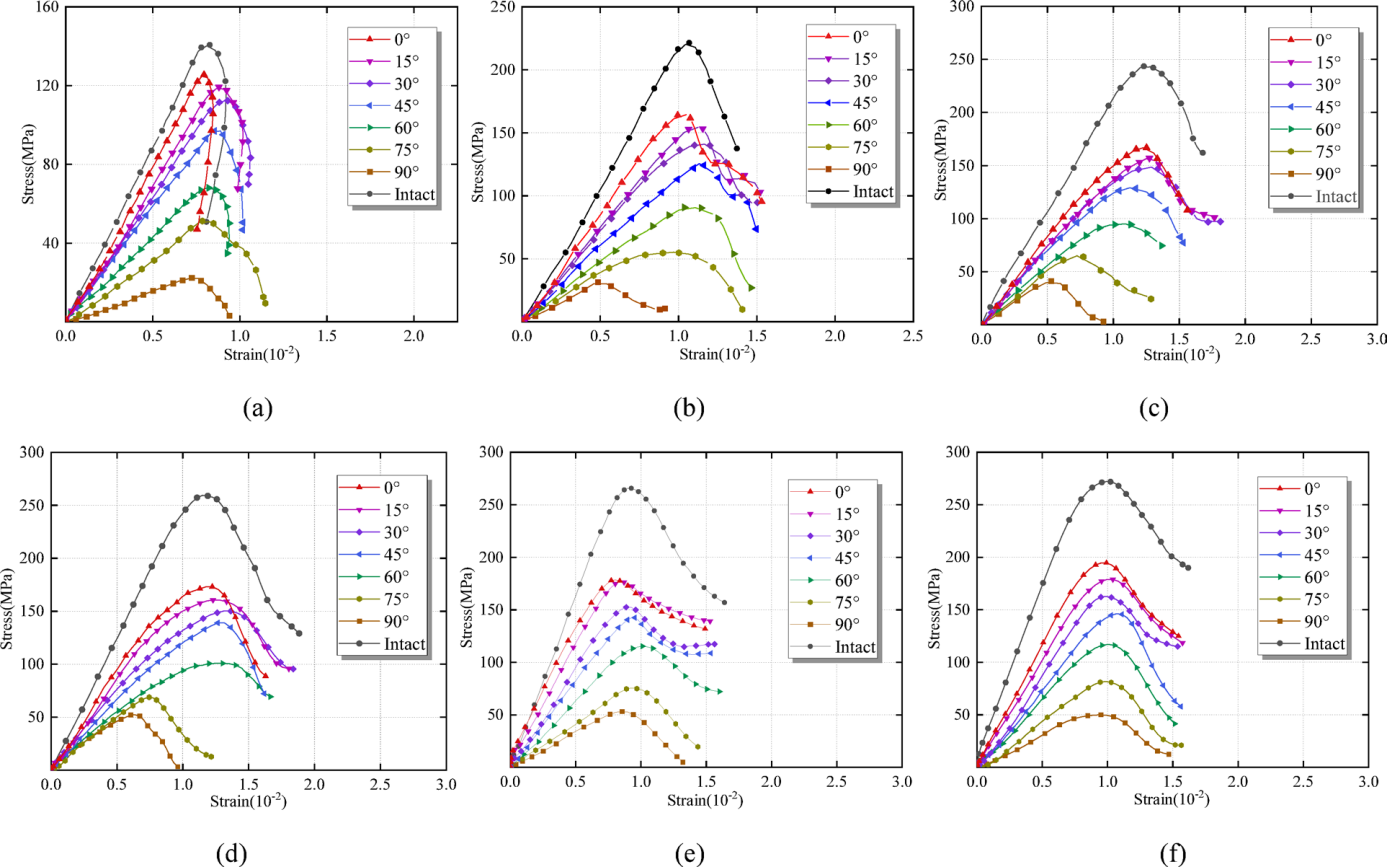
Full stress-strain curves of specimens under different Dynamic load. **(a)** 0.10 MPa. **(b)** 0.15 MPa. **(c)** 0.20 MPa. **(d)** 0.25 MPa. **(e)** 0.30 MPa. **(f)** 0.35 MPa.

Dynamic load and rock angle significantly affect the specimen’s full stress-strain curve (Fig. 4). When the dynamic load is 0.10 MPa, the stress-strain curves of the intact and rock angles of the 0°~60° specimen show an obvious strain rebound phenomenon, indicating that the specimen does not lose its bearing capacity rapidly under the dynamic load. After the failure of the specimen, the block is large and still retains a certain bearing capacity. When the dynamic load is higher than 0.10 MPa, the strain rebound disappears. The strain value of the peak stress of the specimen increases first and then decreases with the increase in the rock angle. The elastic strain energy is released post-peak, and the strain decreases, with the increase in dynamic load, the strain increases continuously. The dynamic load exceeds the specimen’s bearing limit and breaks it into fragments, destroying its bearing capacity.

### Dynamic compressive strength analysis

**Fig. 5:**
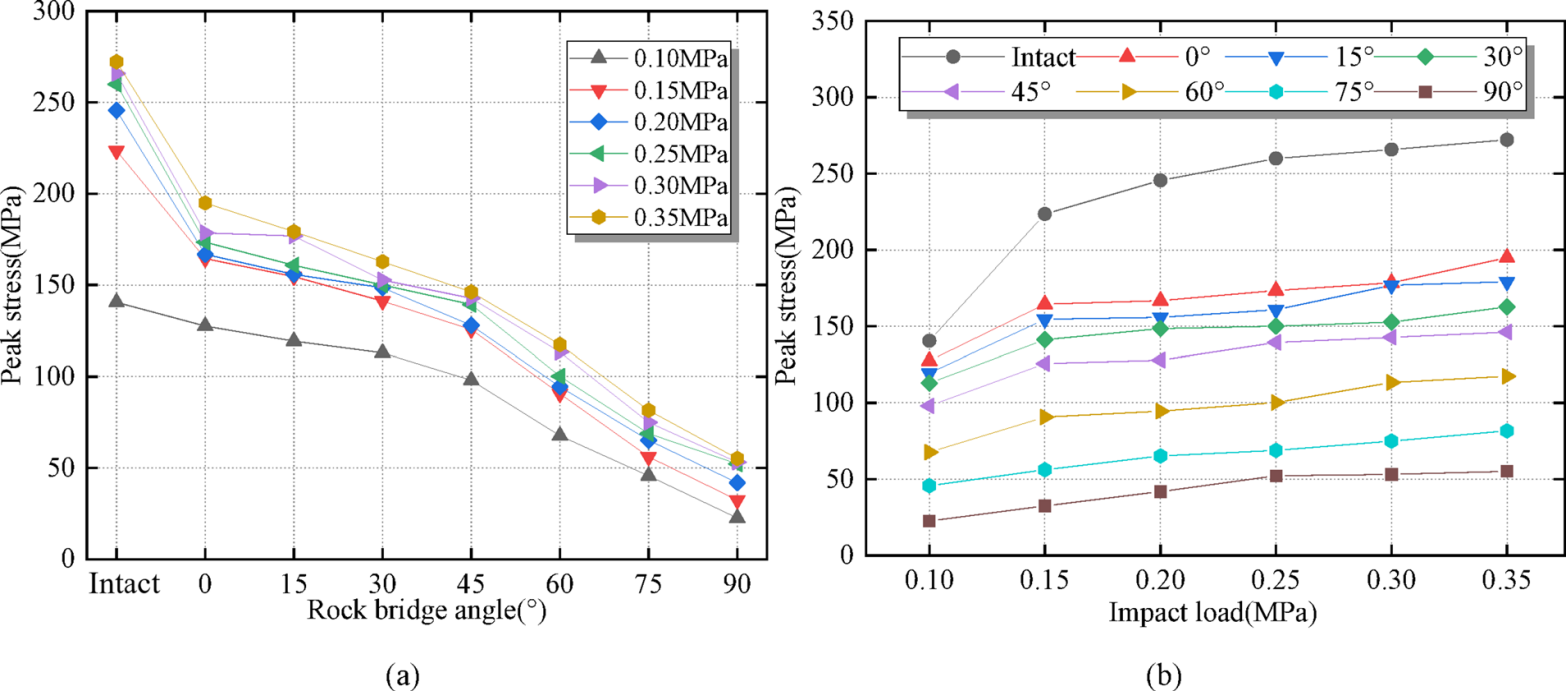
Relationship between peak stress and rock angle and dynamic load. **(a)** Peak stress and rock bridge angle. **(b)** Peak stress and dynamic load.

The peak stress values of the intact specimen are the largest, and the existence of fractures significantly weakens the peak stress of the specimen (Fig. 5(a)). Compared with specimens with different rock angles (0°~90°), the peak stress values are the largest when the rock angle is 0° and the smallest when the rock angle is 90°. This is mainly due to the increase in rock angle; the longer the fracture length, the greater the impact on its structural integrity, resulting in a smaller peak stress. On the other hand, specimen failure is caused by the initiation and propagation of new internal and external cracks. When the rock angle is large, cracks are generated at the fracture tip and connected to each other. This results in a tensile-shear mixed failure that gives the specimen weak impact resistance. Combined with Fig. 5(b), there is a positive correlation between the peak stress values of the specimen and the dynamic load, which indicates that there is a significant rate effect between the two^48,49^. The peak stress variation value of the intact specimen under different dynamic load is 131.7 MPa, while the variation value of the specimens with different rock angles is between 29.8 MPa and 67.4 MPa, which further explains the sensitive peak stress variation characteristics of the intact specimen^50^.

**Table 1:** Stress variation of specimens with different rock Bridge angles under the same dynamic loads.

Dynamic load (MPa)	Stress maximum (MPa)	Minimum stress (MPa)	Stress deterioration Value (MPa)	Degradation percentage (%)
0.10	140.5	22.6	117.9	83.9
0.15	223.5	32.5	191.0	85.5
0.20	245.6	41.8	203.8	83.0
0.25	259.9	52.2	207.7	79.9
0.30	265.8	53.2	212.7	80.0
0.35	272.2	55.2	217.0	79.7

Note: The maximum stress is the rock bridge angle of 0°, and the minimum stress is the rock bridge angle of 90°.

**Table 2:** Stress changes of different dynamic load samples under the same rock Bridge angle.

Rock bridge angle (°)	Stress maximum (MPa)	Minimum stress (MPa)	Stress deterioration value (MPa)	Degradation percentage (%)
0	194.9	127.5	67.4	34.6
15	177.2	119.3	57.9	32.7
30	162.7	112.9	49.8	30.6
45	146.2	98.0	48.2	33.0
60	117.4	67.6	49.8	42.4
75	81.61	45.7	35.9	44.0
90	55.2	22.6	32.6	59.1

Note: The maximum stress is the dynamic load of 0.35 MPa, and the minimum stress is the dynamic load of 0.10 MPa.

According to Table 1, it can be seen that with the increase of dynamic load, the maximum and minimum stress of samples with different rock bridge angles gradually increases, while the deterioration value also increases, and the deterioration percentage is relatively stable between 80% and 85%, indicating that the change in dynamic load has little effect on the deterioration percentage. It can be seen from Table 2 that as the angle of the rock bridge increases, the maximum stress of the sample gradually decreases. The minimum stress also decreases. The larger the angle of the rock bridge is, the more obvious the deterioration effect of the stress is. The deterioration percentage is relatively high. It shows that the dynamic load and the angle of the rock bridge have a significant influence on the stress and deterioration performance of the sample. Especially under the larger dynamic load and the larger angle of the rock bridge, the stress deterioration of the sample is more obvious.

### Crack initiation stress at the fracture tip

**Fig. 6:**
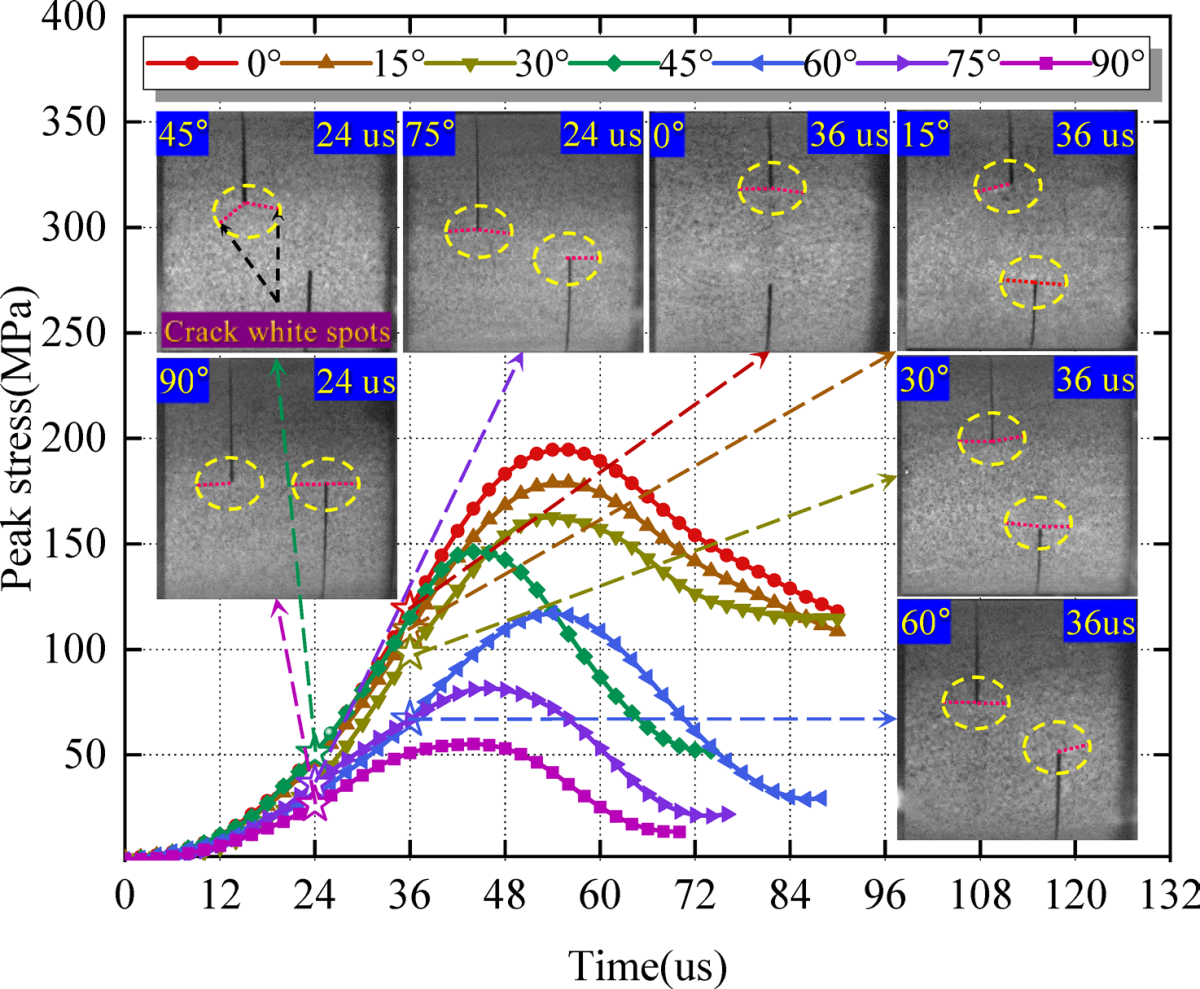
Time history curve of stress and time (0.35 MPa).

In this study, the crack initiation stress at the fracture tip is the critical stress before the crack appears at the fracture tip. The judgment is based on the fact that the white spot of crack initiation during the impact failure process of the specimen, this spot is captured with a high-speed camera, and the corresponding stress at this time is the crack initiation stress, revealer high-speed camera is used(Fig. 6). The maximum frame rate is 1 × 10^5^ FPS, and the minimum time interval is 10 us. According to previous research results^2,51^, the frame number of dynamic mechanical damage image acquisition was usually set in the range of 0.5 × 10^5^ FPS to 1.0 × 10^5^ FPS, and the image interval was between 10 us ~ 20 us. The higher the frame number, the smaller the time interval, the more accurate the crack initiation stress value, but the worse the image clarity.

Before the test, the specimen was tested, and five different acquisition frames were set to be 0.7 × 10^5^ FPS, 0.75 × 10^5^ FPS, 0.80 × 10^5^ FPS, 0.85 × 10^5^ FPS, 0.9 × 10^5^ FPS. During the specimen failure, it was found that the number of acquisition frames was 0.85 × 10^5^ FPS, and the time interval was 12 us. As seen in the image, the white spot of the surface crack of the specimen could be clearly seen (the key factor to determine crack initiation stress), and the expansion scale of the crack was approximately the same. As revealed in the progressive failure image, the white spot of the crack emerges preferentially before the crack. In addition, in the process of high-speed image acquisition, it is necessary to ensure the clarity of the image during the test, accurately select the progressive failure image of the specimen corresponding to the stress-time relationship curve, and ensure that the crack white spot initiation image is selected as the corresponding crack initiation stress image(Fig. 6). Considering space, only a group of specimens with a dynamic load of 0.35 MPa is analyzed for crack initiation stress. Figure 6 shows the time-history curve of stress and time, and the other groups of data are included in Table 3.

**Table 3:** Crack initiation stress and peak stress at fracture tip.

Dynamic load (MPa)		Different rock bridge angle specimens						
		0°	15°	30°	45°	60°	75°	90°
**Time** **(us)**	0.10	108	108	96	96	108	84	84
	0.15	72	72	72	60	72	36	24
	0.20	60	60	36	24	24	24	12
	0.25	48	48	36	24	24	24	12
	0.30	36	36	24	24	24	24	24
	0.35	36	36	36	24	36	24	24
**Crack initiation stress (MPa)**	0.10	61.15	59.23	64.09	47.07	34.61	26.19	11.88
	0.15	78.02	71.68	76.56	63.38	46.02	27.64	15.35
	0.20	87.61	78.87	82.44	63.43	53.01	36.74	19.63
	0.25	91.55	78.79	75.84	72.13	46.52	34.26	30
	0.30	82.37	91.73	78.26	69.17	64.34	36.73	24.77
	0.35	105.70	96.09	81.48	69.20	66.66	42.62	26.24
**Crack initiation stress/peak stress (%)**	0.10	47.96	49.65	56.77	48.03	51.17	57.32	52.61
	0.15	47.46	46.36	54.22	50.46	50.74	49.24	47.27
	0.20	52.52	50.62	55.52	49.59	56.08	56.36	46.93
	0.25	52.80	49.00	50.56	51.78	46.43	49.77	57.43
	0.30	46.15	51.88	51.25	48.47	56.79	48.97	46.60
	0.35	54.23	53.62	50.08	47.33	56.78	52.22	47.54

The crack white spots appear at the fracture tip and extend to both ends; under different dynamic loads and rock angles, there are differences in the time (damage velocity) of crack white spots at the fracture tip of the specimen (Table 3; Fig. 6). Under different dynamic loads, the crack initiation time at the crack tip of the rock angle of 0°~15° is longer. In contrast, the crack initiation time at a 75°~90° rock angle is shorter. At different rock angles, the crack initiation time at the fracture tip of the specimen is longer when the dynamic load is 0.10 MPa. The initiation time is shorter when the dynamic load is 0.30 MPa and 0.35 MPa. Comparing the two, it is found that the specimen’s damage velocity is significantly affected by the dynamic load.

**Fig. 7:**
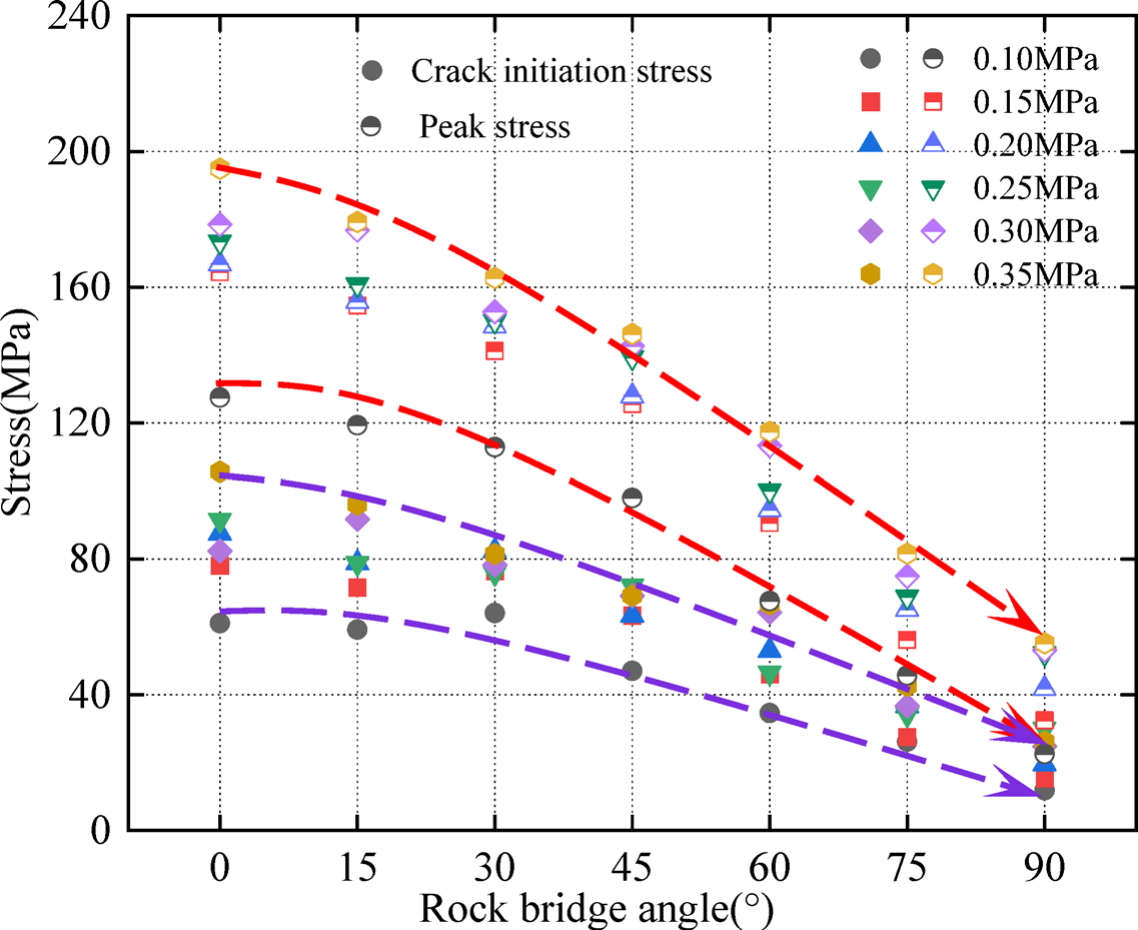
Relationship curves of crack initiation stress and peak stress with rock angle under different dynamic loads.

Crack initiation stress decreases with rock angle increase (Fig. 7). The curves of crack initiation stress, peak stress, and rock angle are approximately the same, and all decrease with rock angle increase. The crack initiation stress is lower than the peak stress, which is consistent with Fig. 5(a). Combined with Table 3, the percentage of crack initiation stress and peak stress is between about 46% and 58%, this is different from Mohsen Nicksiar et al.^52^. It has been found that the percentage of crack initiation stress and peak stress of prefabricated fracture specimens is between 42% and 47% under static conditions. The reason is analyzed that the different loading rates of dynamic and static loading, the loading rate is small under static loading conditions. Under the uniform load applied to the specimen, internal cracks have enough time to initiate and expand. Under dynamic load conditions, the loading speed is large, and the specimen has impact and vibration, resulting in instantaneous failure. The crack inside the specimen does not have enough time to expand, and the crack initiation stress value at the fracture tip is too large^53,54^.

### Fragmentation fractal characteristics

The fragment morphology of the sample was with a rock angle of 45° after impact. It is found that the dynamic load has a strong sensitivity to the degree of fragmentation of the specimen^48^. When the dynamic load is 0.10 MPa, the specimen still maintains a certain degree of integrity, and most are in a blocky dispersion state; with the increase in dynamic load, damage increases(Table 4). For the broken body, the volume decreases significantly, the number increases accordingly, and the failure form changes from crushing to pulverization. To quantitatively characterize the fragmentation degree of the specimen under different dynamic loads, the average fragment size ($\:{d}_{m})$ is introduced to quantitatively describe the fragmentation degree of the specimen after impact compression. The formula is as follows^55^:

10 $$\:\:{d}_{m}=(\sum\:{M}_{i}{\stackrel{-}{d}}_{i}/M)$$

Where: $\:\stackrel{-}{{d}_{i}}$ is the average size of the debris in the sieve size range, mm; $\:{M}_{i}$ is the interval mass of the corresponding fragment, g; and total mass of $\:M$ sieves at all levels, g.

**Table 4:**
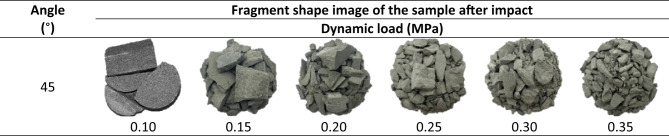
Statistics of fragment morphology after impact crushing of specimens under dynamic load.

**Table 5:**
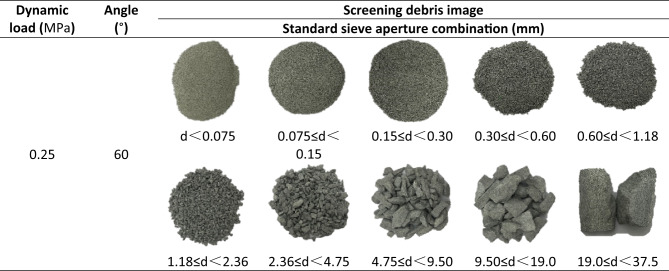
Pictures of broken specimens under dynamic load and standard aperture combination table.

 Taking the specimen with a rock angle of 60° as an example, the dynamic load is 0.25 MPa. The fragmentation distribution is shown (Table 5). According to formula (10), the equivalent average size ($\:{d}_{m})$ is calculated to be 9.78 mm. To further analyze the degree of fragmentation of the specimen, fractal geometry theory is used to analyze the degree of fragmentation. After the test, the specimen fragments were collected and screened. Standard sieves with apertures of 37.5 mm, 19.0 mm, 9.50 mm, 4.75 mm, 2.36 mm, 1.18 mm, 0.60 mm, 0.30 mm, 0.15 mm and 0.075 mm were selected for this combination. The specimens after impact damage were screened, and the crushing blocks at all levels were weighed by a high-precision balance. Previous studies have shown that specimen fragment distribution can be approximated in a power-law form^56^. Rock fragments have self-similarity and fractal characteristics, and their fractal dimension can be determined by Eqs^57,58^. :

11 $$\:{y}_{i}=\frac{M(<{d}_{i})}{M}={\left(\frac{{d}_{i}}{{d}_{max}}\right)}^{3-D}$$

Where:$\:{\:M(<d}_{i})$ is less than the cumulative mass of the fragment size, g; $\:{d}_{max}$ is the maximum fragment size, mm; *D* is the fractal dimension of fragments. Taking the logarithms from both sides of the equation, the formulas (11) and (12) are further simplified. Through the most appropriate fitting of data points, least squares linear regression can be used. The linear fitting line slope is 3-*D*, so the fractal dimension *D* can be determined.

12 $$\:D=3-\lambda\:$$

13 $$\:\lambda\:=\frac{\text{lg}\left[M\right(<{d}_{i})/M]}{\text{lg}\text{(}{d}_{i}\text{/}{d}_{max}\text{)}}$$

**Fig. 8:**
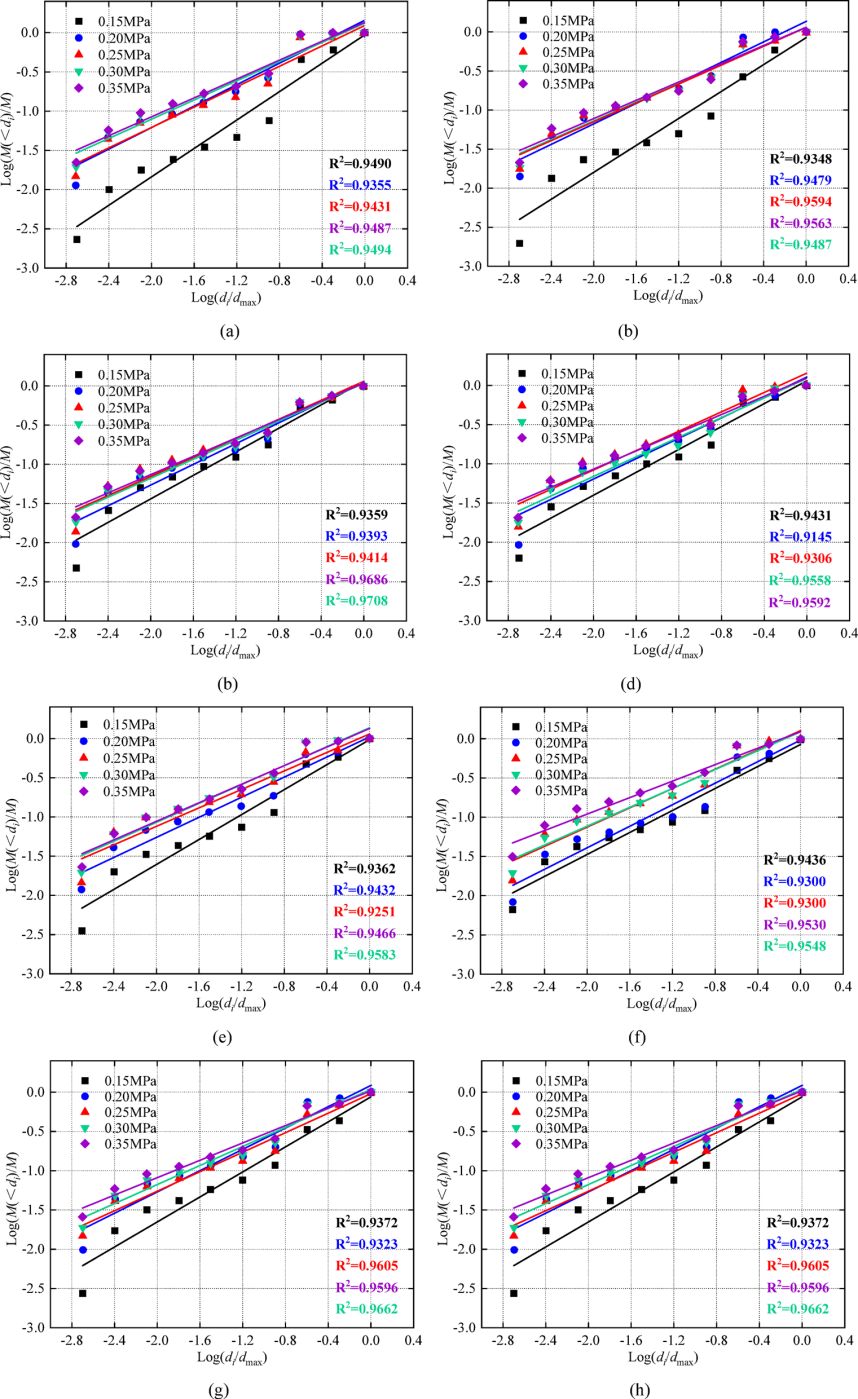
Log$\:(\text{M}\left(<{d}_{i}\right)/\text{M})-\text{L}\text{o}\text{g}({d}_{i}/{d}_{max})$ curve of specimens under different dynamic loads. **(a)** Intact specimen. **(b)** 0°.**(c)** 15°. **(d)** 30°. **(e)** 45°. **(f)** 60°. **(g)** 75°. **(h)** 90°.

By collecting broken specimen blocks, the quality of each sieve particle size is counted. According to formulas (10) and (12), the average fragmentation degree and fractal dimension can be calculated, The influence of dynamic load and rock angle on the degree of fragmentation is analyzed. When the dynamic load is 0.10 MPa, the stress-strain curve of the specimen shows an obvious strain rebound phenomenon. The actual specimen has not been completely destroyed, and the broken block is so large that it is not analyzed; the specimen fragments are irregular. Therefore, the fractal dimension and the average fragment size are the characteristic quantities describing fragment size, and quantitatively characterize the relationship between the fragment mass and the equivalent size and damage degree of the specimen. According to the test data, it can be seen that the fractal dimension changes in the range of (2.094 ~ 2.475). There is a significant logarithmic correlation between the cumulative mass of the particle size under the sieve and the diameter of the standard sieve. Using the linear fitting Eq. (14), the Log(M(< d_i_)/M)-Log(d_i_/d_max_) curves of the specimens under different dynamic loads are drawn (Fig. 8). The *R*^*2*^ of the fitting line of the data points under each working condition is between 0.9145 and 0.9708, and the correlation coefficient is high, indicating that the specimen has obvious self-similarity and fractal characteristics.

14 $$\:y=ax+b$$

The slope of the fitting curve decreases with dynamic load (Fig. 8). In contrast, the fractal dimension grows gradually, indicating that the fragmentation degree increases with dynamic load. Crushing after impact has obvious fractal characteristics. The more the number of fragments, the greater the value of the fractal dimension, the smaller the size, and the greater the degree of fragmentation. The values of related parameter coefficients *a* and *b* are shown in Table 6.

**Table 6:** Correlation coefficient.

Dynamic load	Coefficient	Rock bridge angles(°)							
		Intact	0	15	30	45	60	75	90
0.15	*a*	−0.027	−0.073	0.058	0.060	−0.014	−0.072	−0.060	−0.022
	*b*	0.906	0.862	0.750	0.730	0.796	0.701	0.796	0.717
0.20	*a*	0.154	0.136	0.049	0.101	0.015	−0.012	0.080	−0.008
	*b*	0.684	0.656	0.661	0.650	0.641	0.687	0.678	0.667
0.25	*a*	0.088	0.064	0.049	0.145	0.058	0.100	−0.017	0.113
	*b*	0.650	0.603	0.607	0.618	0.589	0.615	0.621	0.666
0.30	*a*	0.114	0.056	0.032	0.079	0.130	0.088	0.032	0.037
	*b*	0.612	0.598	0.606	0.626	0.603	0.602	0.606	0.570
0.35	*a*	0.129	0.058	0.030	0.085	0.124	0.089	0.017	0.068
	b	0.602	0.585	0.584	0.580	0.592	0.525	0.553	0.575

**Fig. 9:**
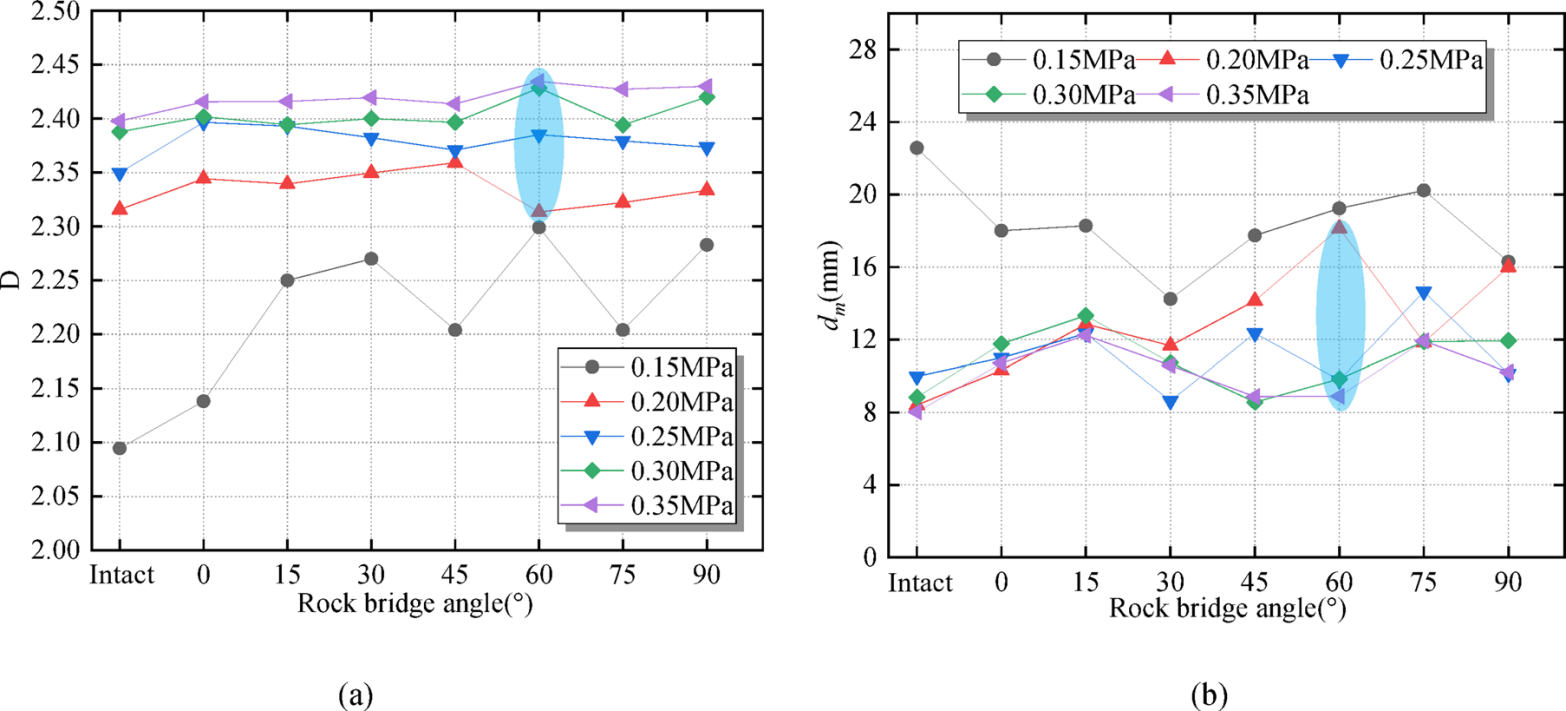
Relationship curves between fractal dimension, average fragment size and rock bridge angles. **(a)** Fractal dimension and rock bridge angle. **(b)** Average fragment size and rock bridge angle.

Under the same dynamic load, the difference rates between the maximum and minimum fractal dimension values of different rock angle specimens are 7.5%, 2.0%, 2.0%, 1.4% and 0.9%, respectively. When the dynamic load is 0.15 MPa, the fractal dimension change value is significantly affected by the rock angle, while the influence is weak at 0.35 MPa, indicating that the larger the dynamic load is, the weaker the fractal dimension of the specimen is affected by the rock angle (Fig. 9(a)). This is because with the increase in dynamic load, the instantaneous load of the specimen increases. In contrast, fracture microstructure has no significant effect on the damage degree. Combined with Fig. 9(b), the average fragment size shows similar characteristics to the fractal dimension. However, when the dynamic load is 0.15 MPa, the average fragment size of the specimen is opposite to the fractal dimension. When the rock angle is 60°, the fractal dimension and the average fragment size show more sensitive variation characteristics.

### Crack propagation

**Fig. 10:**
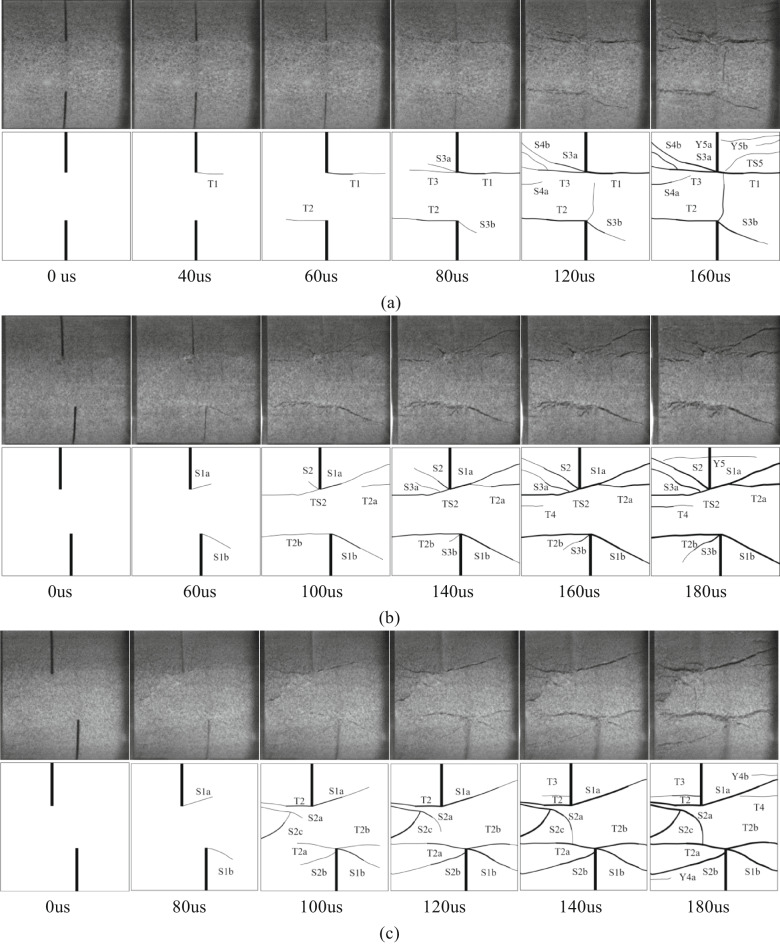

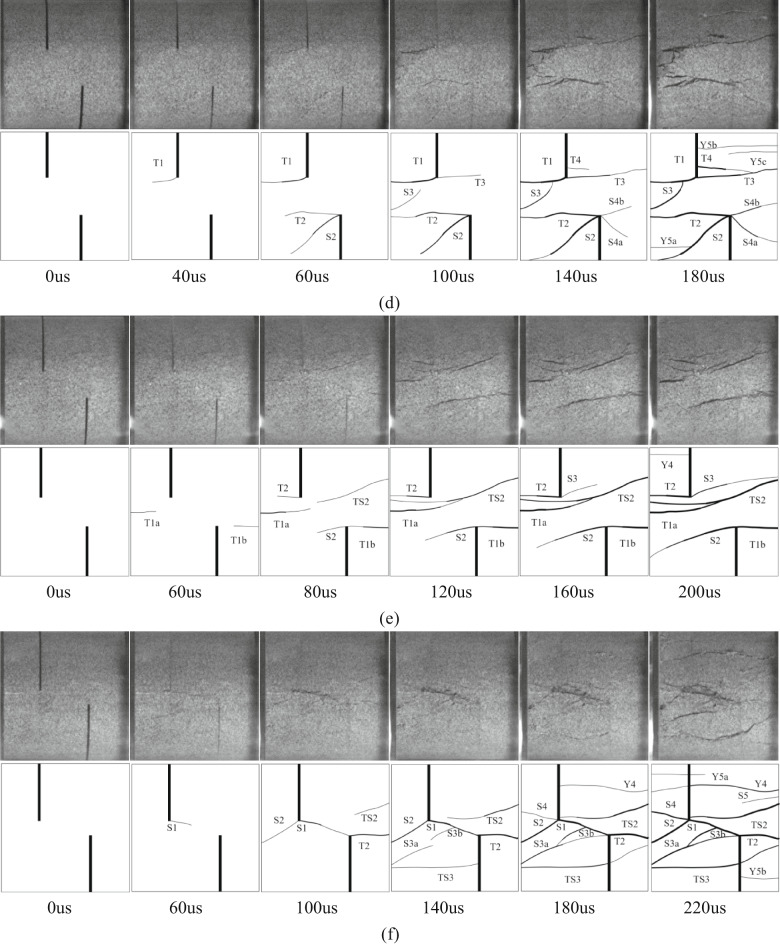

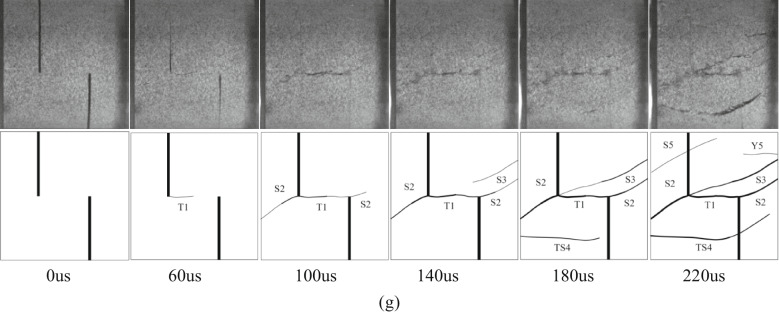
Failure process and sketch of specimens with different rock bridge angles under dynamic load (0.25 MPa). **(a)** YS-20-0°. **(b)** YS-20-15°. **(c)** YS-20-30°. **(d)** YS-20-45°. **(e)** YS-20-60°. **(f)** YS-20-75°. **(g)** YS-20-90°.

In the Fig. 10, T represents wing cracks and anti-wing cracks with tensile properties, S represents cracks with shear properties, TS represents tensile-shear mixed cracks, Y represents far-field cracks, and numbers represent the time sequence of cracks. The letters (a, b, c) after the numbers are used to distinguish the same type of crack that appears at the same time. The direction of dynamic load is left to right. Taking sandstone specimens with rock angles of 0°, 15°, 30°, 45°, 60°, 75° and 90° under 0.25 MPa impact pressure as an example, the crack propagation of specimens with different rock angles under dynamic load was analyzed.

Considering the length of the article, taking the sample with a rock bridge angle of 75° as an example, the crack propagation process of the sample with different rock bridge angles under dynamic load is described, as shown in Fig. 10.

At 60 us, a shear crack S1 appeared between the two cracks, which connected the ends of the cracks to form a rock bridge. At 100 us, tensile crack T2 appeared at the end of the right crack in the loading direction. It extended to the right end boundary of the sample. Shear crack S2 appeared in the left crack of the sample, and the tensile-shear mixed crack TS2 appeared at the right end. At 140us, the tensile-shear mixed crack TS2 continues to expand to the left crack tip and TS3 extends to the middle of the right crack. The secondary shear cracks S3a and S3b appear at the left end of the sample and in the middle of the fractured rock bridge. At 180 us, the shear crack S4 appeared at the end of the left crack. With the further development of the crack. At 220 us, the far-field cracks Y5a and Y5b appeared at the upper left and lower right of the sample, the tensile shear crack TS3 appeared at the lower end of the sample, and the tensile crack Y4 appeared at the upper end. At the same time, the shear crack S5 appears at the right end of the sample, and the shear crack S1 evolves into a fracture zone and produces rock powder. The sample fails tensile-shear mixed type, but the shear crack dominated the failure.

Under dynamic load, the crack tip will first produce stress concentration and crack along the axial direction, and then the rock bridge is connected. The macroscopic cracks of 0°, 15°, 30° and 45° specimens are mainly tensile cracks. The macroscopic cracks of 60°, 75° and 90° specimens are shear cracks.

### Failure mode

**Fig. 11:**
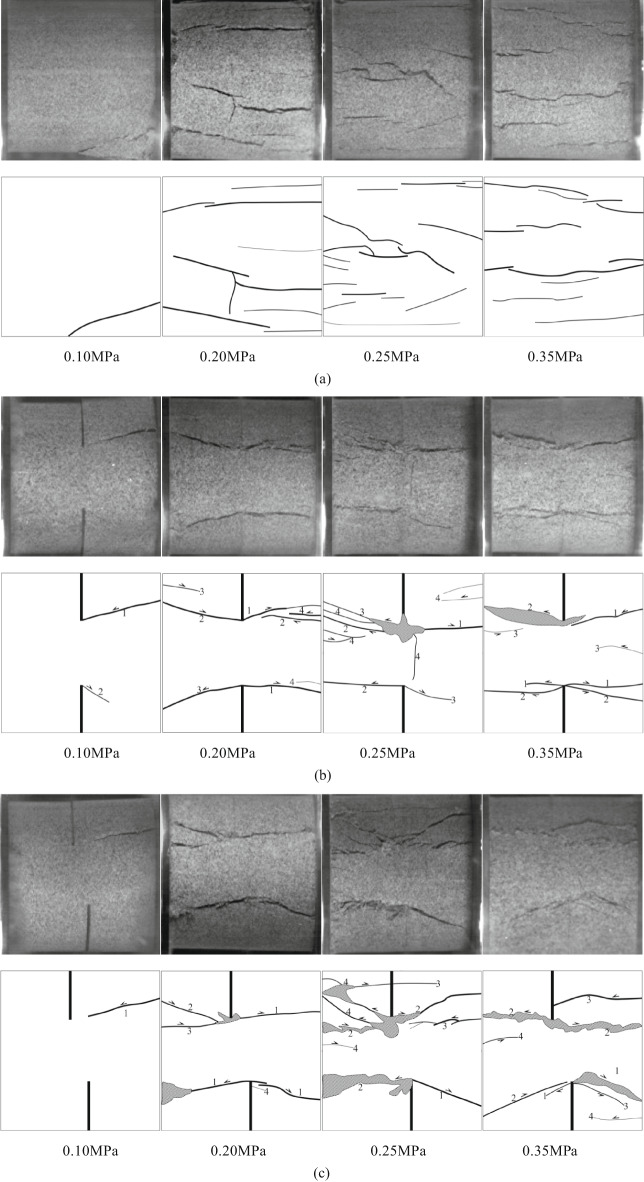

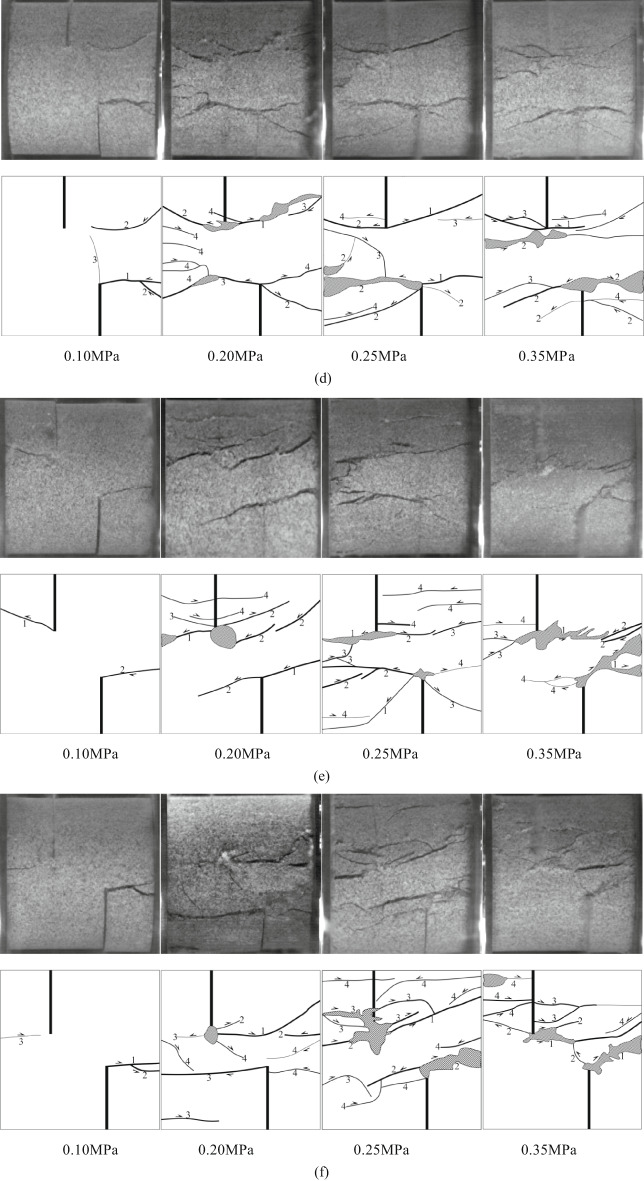

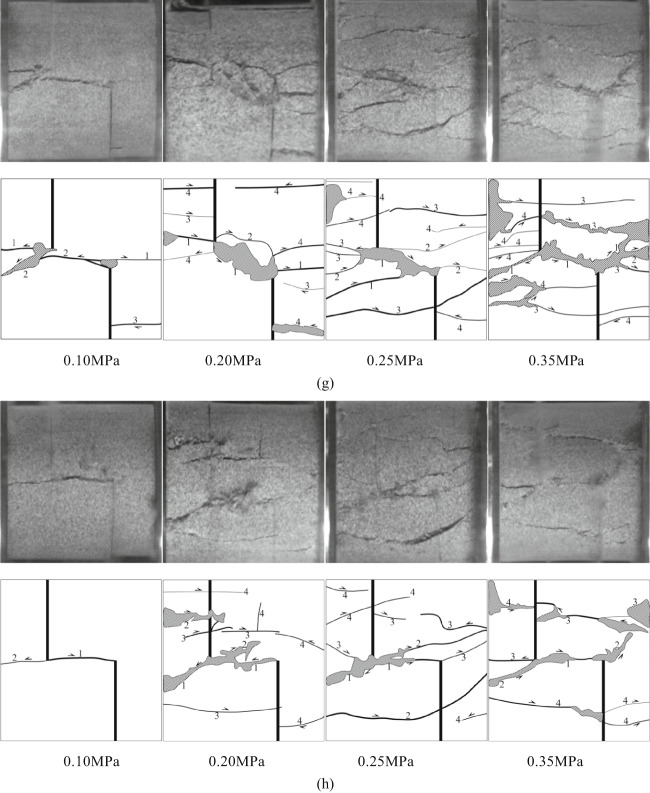
Failure modes of intact and different rock knocking angle sandstone specimens under dynamic load. **(a)** Intact specimen. **(b)** YS-20-0°. **(c)** YS-20-15°. **(d)** YS-20-30°. (e) YS-20-45°. (f) YS-20-60°. (g) YS-20-75°. (h) YS-20-90°.

**Fig. 12:**
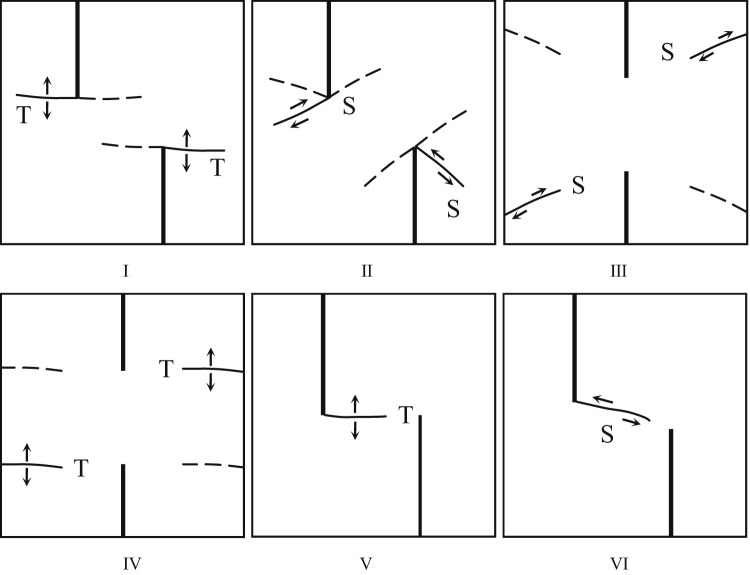
Various typical crack types identified in different rock corner samples. T-type tensile crack, S-type shear crack (dynamic load direction: from left to right).

Based on the above crack propagation characteristics, Fig. 11 shows the final failure mode and sketch map of the specimen. Combined with the failure image in Fig. 10, six kinds of crack aggregation forms with different trajectories and propagation mechanisms can be identified, as shown in Fig. 12.

According to the high-speed camera images, nine types of cracks are analyzed and summarized, and classified according to the initiation and propagation mechanism of cracks, as shown in Fig. 12. Among them, three types of cracks are tensile cracks (I, IV and V), and three types are shear cracks (II, III and VI). The detailed analysis of each crack type is as follows:

#### Type I:

The tensile wing crack and the anti-wing crack start at or near the inner tip of the crack, and then expand independently along the loading direction. As the dynamic load increases, the frequency of this crack gradually increases. This type of crack is common under large dynamic loads.

#### Type II:

The shear anti-wing crack starts at the inner end of the existing crack and extends to the edge of the specimen at a certain angle in the axial stress direction. With the increase in dynamic load, the frequency of occurrence is increasing. The formation of this type of crack is closely related to the increase in dynamic load.

#### Type III:

The shear crack starts at the boundary of the sample and expands into the interior of the sample, and the occurrence is random.

#### Type IV:

The tensile crack starts at the sample boundary and extends to the crack tip. When the dynamic load is higher than 0.20 MPa, the specimen generally produces this type of crack. This indicates that a higher dynamic load is more likely to cause this type of crack.

#### Type V:

The tensile crack starts at the tip of the internal crack along the loading direction and extends to the other tip along the loading direction. This type of crack is common in samples with a rock bridge angle of 90°. This is not related to the size of the dynamic load, but also related to the form of the crack.

#### Type VI:

The starting point and propagation direction of the shear crack are similar to those of the VII type, which appears in the sample with a rock bridge angle of 75°. The difference between the V-type crack is that this type of crack does not appear under low dynamic load. The reason is that when the dynamic load is small, the sample is not completely destroyed, so no such crack is generated.

**Table 7:** Crack types of sandstone samples with different rock angles.

dynamic load	Sample number	Crack propagation types					
		Ⅰ	Ⅱ	Ⅲ	Ⅳ	Ⅴ	Ⅵ
0.10 MPa	YS-20-0°		√^①^				
	YS-20-15°			√^①^			
	YS-20-30°	√^①^		√			
	YS-20-45°	√^①^			√		
	YS-20-60°	√^①^			√		
	YS-20-75°	√^①^	√				
	YS-20-90°	√				√^①^	
0.20 MPa	YS-20-0°	√^①^		√			
	YS-20-15°	√^①^		√			
	YS-20-30°		√	√^①^			
	YS-20-45°		√	√^①^			
	YS-20-60°	√^①^			√		
	YS-20-75°	√^①^			√		√
	YS-20-90°		√		√	√^①^	
0.25 MPa	YS-20-0°	√^①^	√		√		
	YS-20-15°	√	√^①^		√		
	YS-20-30°	√	√^①^		√		
	YS-20-45°	√	√^①^		√		
	YS-20-60°	√^①^	√	√	√		
	YS-20-75°	√		√	√		√^①^
	YS-20-90°		√		√	√^①^	
0.35 MPa	YS-20-0°	√^①^	√		√		
	YS-20-15°	√	√^①^		√		
	YS-20-30°	√	√^①^	√	√		
	YS-20-45°	√	√^①^	√	√		
	YS-20-60°	√^①^	√	√	√		
	YS-20-75°	√		√	√		√^①^
	YS-20-90°	√	√		√	√^①^	

Note: T-type tensile cracks; S-shaped shear cracks(dynamic load direction: from left to right);√ indicates that this type of crack appears; ① indicates that the crack first appears.

According to Table 7, it can be seen that there are obvious differences in the types of crack propagation of sandstone samples under different dynamic loads. With the increase of dynamic loads, the type of crack propagation gradually tends to be complex, from a single crack to a combination of multiple types. Under low load (0.10 MPa, 0.20 MPa), the cracks mainly show a small amount of type I, type II or type III expansion. The distribution is relatively dispersed, and the number of cracks is limited. When the load increases to 0.25 MPa and above, the crack types begin to show a trend of simultaneous development of multiple types. The phenomenon of crack interaction and penetration is more significant, indicating that impact strength is the key factor to control crack complexity and diversification.On the other hand, different rock bridge angles also have a significant effect on crack propagation modes. When the angle of a rock bridge is 0°~30°, the crack types are mainly type I and type II. It is easier to form a penetrating crack in the direction of the principal stress under load. When the angle of a rock bridge is 45°~75°, the types of crack are more diverse. The frequency of type III and V cracks increases, showing the composite effect of shear and tension. When the angle is 90°, the cracks tend to be concentrated on types IV and type VI. It is easy to produce obvious crushing and splitting characteristics at high loads. On the whole, crack propagation laws are controlled by dynamic load and rock angle. The dynamic loads of 0.10 MPa and 0.20 MPa and the rock bridge angle of 0°~30° are mainly single cracks. The dynamic loads of 0.25 MPa and 0.30 MPa and the rock bridge angle of 45°~90° tend to be complex multi-crack propagation.

## Energy dissipation law

### Energy dissipation

**Fig. 13:**
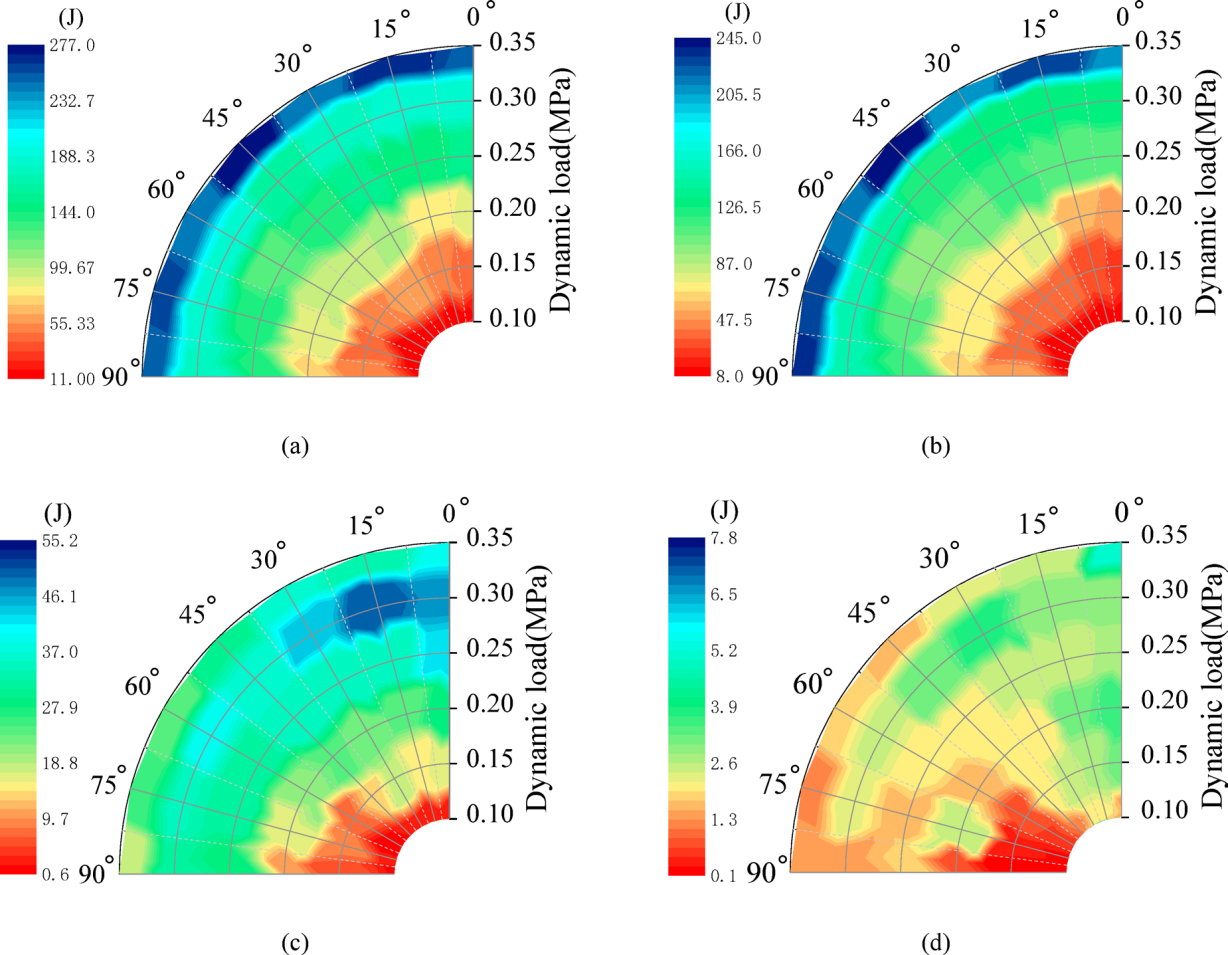
The energy relationship fan diagram of rock angle samples under different dynamic loads. **(a)** incident energy. **(b)** reflection energy. **(c)** transmitted energy. **(d)** dissipated energy.

Energy dissipation is characterized by crack propagation and failure processes. Based on dynamic energy theory, incident energy, reflection energy, transmission energy, dissipation energy, energy dissipation rate, and energy dissipation density are obtained by formulas (3)~(8). According to the test data, the energy relationship fan diagram of rock angle samples under different dynamic loads (Fig. 13).

The incident energy and reflection energy increase with an increase in dynamic load, and there is a significant rate effect between them. Under the same dynamic load, the incident energy and reflection energy of specimens with different rock angles are less affected by the rock angle. This is shown in Fig. 13(a) and (b). The dissipation energy increases first and then decreases with the increase in dynamic load. When the dynamic load is 0.30 MPa, the dissipation energy of 0°, 15° and 30° specimens is larger. In contrast, the dissipation energy of 75° and 90° specimens is smaller, as shown in Fig. 13(c). When the dynamic load is 0.35 MPa, the transmission energy of the specimen decreases gradually with the increase in the rock angle. The transmission energy of the 0° specimen is the largest, as shown in Fig. 13(d). In summary, the higher the dynamic load, the increased the incident energy, and the degree of fragmentation is significant. The specimen fails to convert most of the absorbed energy into the kinetic energy of fragment ejection. Therefore, the ratio of dissipated energy to incident energy is closely related to fragmentation degree.

### The influence of rock angle on energy dissipation under different dynamic loads

**Fig. 14:**
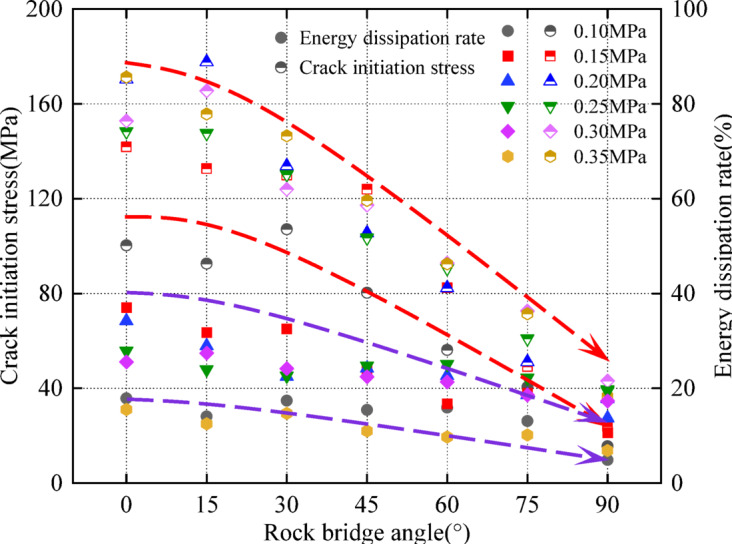
Relationship between initiation stress, energy dissipation rate and rock angle under different dynamic loads.

The variation trend of crack initiation stress and energy dissipation rate is approximately the same; this decreases with the increase in rock angle. The energy dissipation rate of the specimen with a rock angle of 90° is the smallest (Fig. 14). This indicates that the 90° specimen is more prone to instability. This is because the 90° specimen fracture length is longer; this weakens the integrity of the structure and reduces the ability of the specimen to resist impact, which is consistent with Fig. 5(a). Combined with Fig. 7, under different dynamic loads and rock angles, the overall trend of peak stress, crack initiation stress, and energy dissipation rate of the specimen is approximately the same. This indicates that the rock angle has a significant effect on peak stress, crack initiation stress, and energy dissipation rate.

**Fig. 15:**
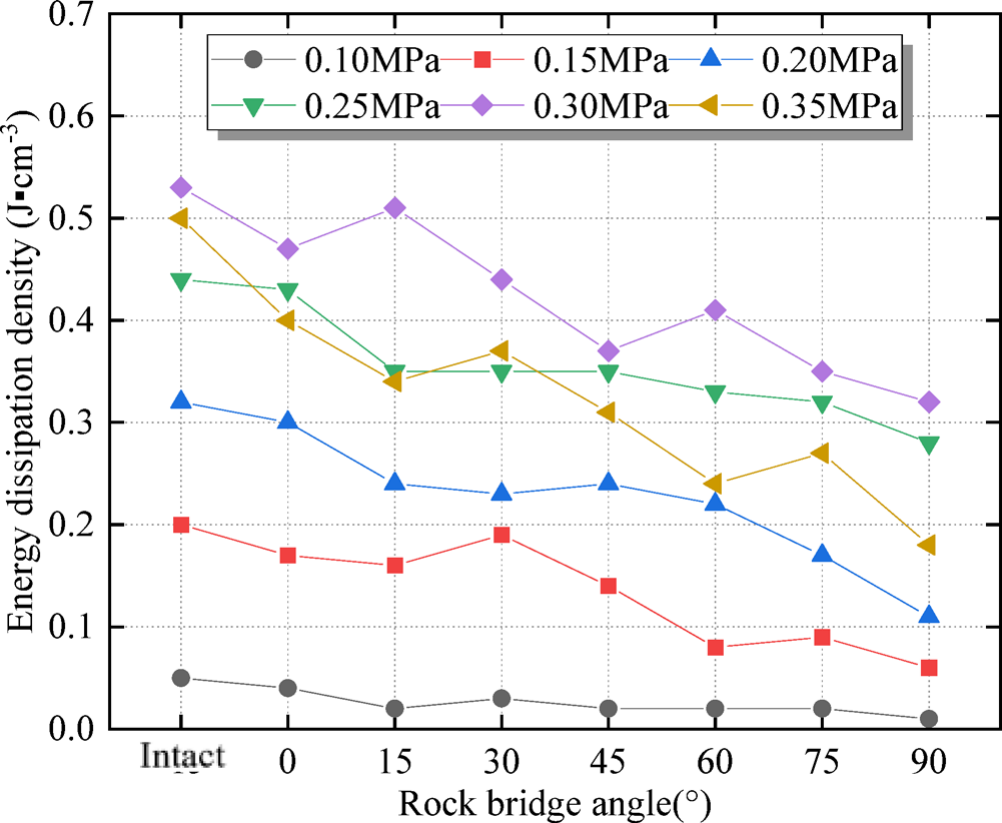
Relationship between energy dissipation density and rock angle.

It can be seen from Fig. 15 that the energy dissipation density increases with dynamic load increases, while the energy dissipation density decreases as a whole due to the influence of rock angle, which corresponds to Fig. 14. With the increase in dynamic load, crack initiation and propagation inside and outside the specimen will consume more energy. From the perspective of practical engineering, when encountering multi-fractured rock mass with multi-fracture and fracture tip rock bridge angle of 90°, structural adjustment should be carried out in time, and the fracture should be filled to increase its integrity. At the same time, to release accumulated energy and reduce impact risk, pressure relief can be used.

**Fig. 16:**
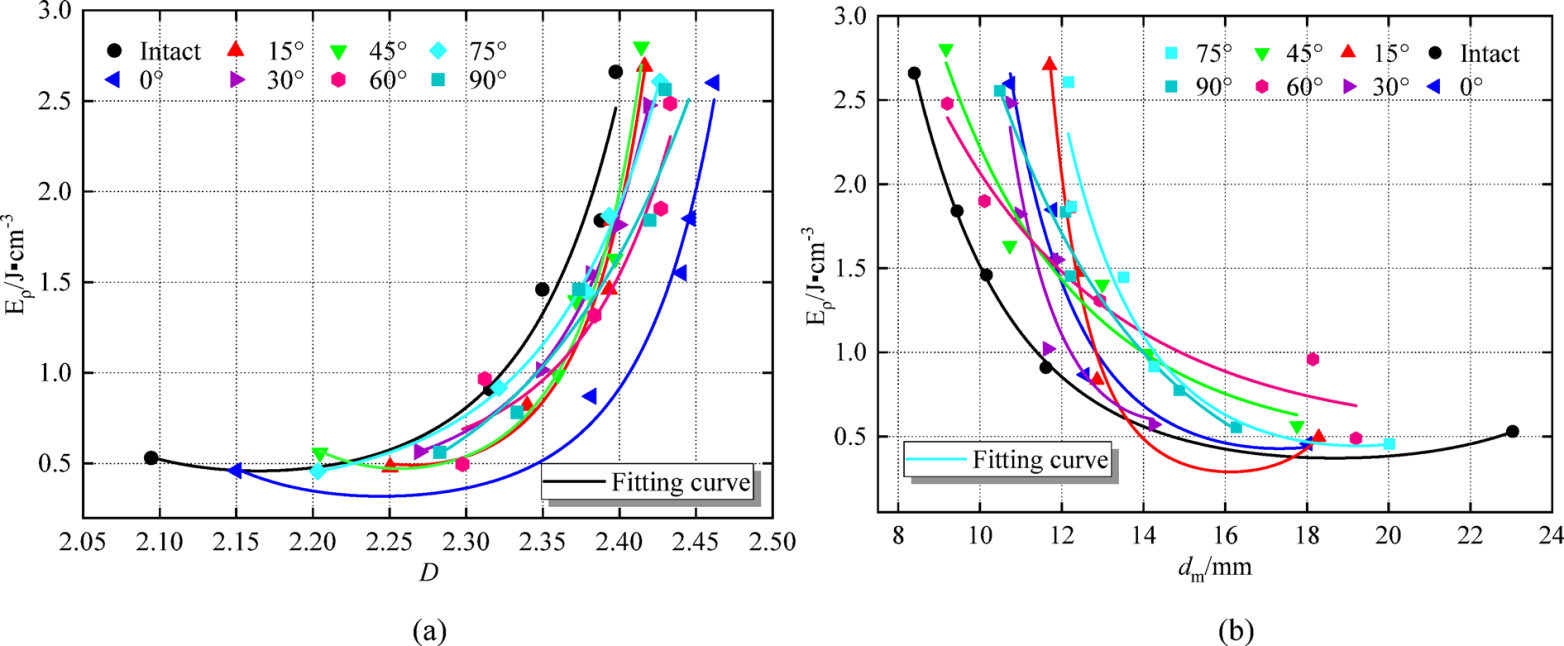
Curve of energy dissipation density versus fractal dimension and average fragment size of specimens. **(a)** Energy dissipation density and fractal dimension. **(b)** Energy dissipation density and average fragment size.

In summary, according to the energy dissipation density, fractal dimension, and average fragment size, the exponential function relationship formula (15) is satisfied. The energy dissipation density, fractal dimension, and average fragment size scatter plots of different rock angle specimens are fitted (Fig. 16), and the correlation coefficient values are listed in Table 8.

15 $$\:{y}_{i}={e}^{(a+bx+c{x}^{2})}$$

**Table 8:** Correlation coefficients of exponential relationship curves.

Physical quantity	Angle (°)	Coefficient			
		*a*	*b*	*c*	*R* ^2^
*D*	/	144.0	−133.9	30.9	0.9046
	0	272.6	−246.3	55.4	0.9605
	15	373.6	−330.5	73.0	0.9559
	30	155.9	−142.8	32.5	0.9930
	45	350.5	−311.5	69.0	0.9160
	60	152.6	−137.8	31.0	0.9002
	75	86.5	−82.6	19.5	0.9904
	90	−23.5	10.7	0.3	0.9514
*d*_m_	/	6	−0.7	0.02	0.9980
	0	2.7	−1	0.04	0.9012
	15	8.2	−4	0.1	0.9743
	30	5.4	−3	0.09	0.8954
	45	4	−0.4	0.01	0.9183
	60	3	−0.3	0.006	0.9087
	75	2.7	−1	0.03	0.8972
	90	4	−0.3	−0.05	0.9593

It can be seen from Table 8; Fig. 13 that the energy dissipation density increases with the fractal dimension. It decreases with average fragment size increase. The *R*^*2*^ of the fitting line of the data points under each working condition is between (0.9002 ~ 0.9904) and (0.8954 ~ 0.9980), and the fitting coefficient is high. Under different dynamic loads and rock angles, the fractal dimension and the average crushing size have an exponential relationship with the energy dissipation density. With an increase in dynamic load, the fractal dimension increases, the dissipation energy increases, and the energy used for crack initiation, propagation, and re-propagation increases. The damage degree of the specimen is more serious, which is manifested by the increase in the number of small particle size fragments; this increases the fractal dimension. In addition, the average fragment size decreases, and the degree of damage increases. The two exponential curves show opposite relationships.

## Discussions

With the increase in dynamic load, the damage degree of the specimen at the same rock angle grows gradually (Figs. 8 and 10). Compared with actual underground engineering, when the dynamic load gradually increases, the damage degree of the fractured surrounding rock mass increases. This increases the risk of a rock bursts. When the dynamic load is small enough, the damage degree of the broken surrounding rock mass decreases, and the risk of rock burst decreases. Therefore, in rock burst disaster prevention and control, the dynamic load size should be evaluated in advance. When the dynamic load in this area is large, pressure relief of fractured surrounding rock should be carried out in advance; this will increase the dissipation energy.

The existence of prefabricated fractures significantly weakens the peak stress of the specimen. When the rock angles are 0 and 90, the peak stress values are both the largest and the smallest. There is a significant rate effect between the rock angle and the dynamic load, and dynamic load and rock bridge angle have a significant impact on the stress and deterioration performance of the samples, especially under higher dynamic load and larger rock bridge angle, where the stress deterioration of the samples is more pronounced (Figs. 5; Tables 1 and 2). Corresponding to the actual project, when the rock angle of the fractured surrounding rock mass is 0, the dynamic failure is not easy to occur. Dynamic failure occurs more frequently at 90° rock angles. Therefore, the risk of a rock burst disaster increases when the fractured surrounding rock mass has a large rock angle. In the anti-impact design of underground structures, geological factors on rock angles should be fully considered. When there is a large rock angle in the fractured surrounding rock mass, micro-cracks should be grouted to change structural integrity and adjust the stress propagation path.

There are significant differences in the crack initiation time (damage velocity) of the sandstone fracture tip under different dynamic loads and rock angles, and different dynamic loads have a significant effect on sandstone damage velocity (Table 3; Fig. 6). For actual underground engineering, low dynamic load causes crack initiation time at the fracture tip to be relatively slow, while high dynamic load is the opposite. With roof cutting and pressure relief, the dynamic load source at a certain position on the roof can be eliminated in order to reduce its impact risk on the fractured surrounding rock mass. After the adjustment of roof cutting and pressure relief, the spatial relationship between the dynamic load source and the crack position changes. The risk of a rock burst disaster is greatly reduced.

## Conclusion

(1) The strain value of the peak stress of the specimen increases first and then decreases with the increase in the rock angle. The peak stress values of the intact specimen are the largest and show the most sensitive characteristics. Prefabricated fractures significantly weaken peak stress; when the rock angles are 0° and 90°, the peak stress values are the maximum and minimum. As the dynamic load increases, the maximum stress, minimum stress, and deterioration value of samples with different rock bridge angles gradually increase, while the deterioration percentage remains relatively stable, indicating that the dynamic load has little effect on the deterioration percentage. As the rock bridge angle increases, the maximum and minimum stresses of the sample gradually decrease, and the larger the rock bridge angle, the more pronounced the deterioration effect. The crack propagation of sandstone samples is mainly controlled by dynamic load and rock bridge angle. When the dynamic load is 0.10 MPa and 0.20 MPa, and the rock bridge angle is 0°~30°, cracks are of a single type, with limited number and dispersed distribution. When the dynamic load exceeds 0.25 MPa and the rock bridge angle is 45°~90°, cracks become more diverse, with shear-tensile composite failure occurring easily, accompanied by significant crack penetration and fragmentation. The dynamic load determines the complexity of cracks, while the rock bridge angle influences the distribution of crack types.

(2) There is a significant rate effect between the incident energy, reflected energy and dynamic load. With an increase in dynamic load, energy dissipation increases first and then decreases. The energy dissipation of 0° and 15° specimens is larger, while the energy dissipation of 90° specimens is smaller. The energy dissipation rate decreases with the increase in rock angle. The energy dissipation rate and fractal dimension both increase with the increase in dynamic load. The energy dissipation density increases with the increase in dynamic load, and the influence of rock angle shows a decreasing trend. While energy dissipation density has an opposite relationship with the exponential curve of fractal dimension and average fragment size. When the dynamic load is 0.15 MPa, the rock angle has a significant influence on the change in fractal dimension. This is opposite to the average fragment size. The average fragment size and the fractal dimension show similar variation characteristics. Especially when the rock angle is 60°, the fractal dimension and the average fragment size show a more sensitive response to dynamic load changes.

(3) The crack initiation stress at the fracture tip gradually decreases with rock angle increase and is lower than the peak stress. The percentage of crack initiation stress and peak stress is between about 46% and 58%. Under different dynamic loads and rock angles, there are significant differences in the crack initiation time (damage velocity) at the fracture tip of the specimen. By comparison, it is found that dynamic load has a significant influence on the damage velocity of the specimen. The overall trend of peak stress, initiation stress, and energy dissipation rate under different dynamic loads and rock angles is approximately the same. This indicates that there is a certain correlation between a variety of dynamic loads and rock angles and peak stress, initiation stress, and energy dissipation rate.

## Data Availability

The datasets used and analyzed in this study can be provided by the authors Qinghai Zhang, Xiaoliang Xu, and Lihua Wu according to reasonable requirements.
